# Applying nanoemulsions produced from the essential oil mixture of citronella and *Coleus Amboinicus* to preserve minced chicken meat

**DOI:** 10.1371/journal.pone.0339984

**Published:** 2026-01-05

**Authors:** Tan Phat Vo, Nguyen Van Nhi Le, Tan Triet Tcheng, Le Song Tu Pham, Tong Minh Quan Truong, Ngoc Bao Vy Nguyen, Dinh Quan Nguyen

**Affiliations:** 1 Laboratory of Biofuel and Biomass Research, Faculty of Chemical Engineering, Ho Chi Minh City University of Technology (HCMUT), Ho Chi Minh City, Vietnam; 2 Vietnam National University Ho Chi Minh City, Thu Duc City, Ho Chi Minh City, Vietnam; Prince of Songkla University, THAILAND

## Abstract

This study investigated the preservative potential of four essential oils (EOs): citronella (Ci), *Litsea cubeba* (Lc), *Coleus amboinicus* (Ca), and kaffir lime (Kl) for minced chicken (MC). Their major constituents (geraniol, citral, isobornyl acetate, and D-limonene, respectively) were identified, and their antibacterial and antioxidant capacities were evaluated. A mixture of Lc and Ca displayed the lowest MIC against *L. plantarum* (2 µL/mL), while citronella showed the highest activity against *E. coli* (6 µL/mL). Synergy analysis indicated that the CiCa combination produced the strongest antibacterial response and exhibited the highest antioxidant capacity. Based on this dual bioactivity, the CiCa mixture was formulated into a nanoemulsion and incorporated into a chitosan film to develop a nanoemulsion phase inversion chitosan film (NPICF). When applied to MC and stored for 10 days, NPICF significantly delayed spoilage, maintaining a low total viable count (3.92 log_10_ CFU/g) and total volatile basic nitrogen (1.59 mg/100 g), while preserving texture (hardness: 663 g; cohesiveness: 0.376). These findings demonstrate that NPICF is a promising natural strategy for extending the shelf-life and maintaining the quality of minced chicken.

## 1 Introduction

Bacterial contamination remains a significant threat to global food security and public health, serving as the primary cause of food spoilage and numerous foodborne illnesses. Conventional synthetic preservatives, such as sodium nitrite, benzoates, and potassium sorbates, have been used for a long time to mitigate this issue. However, their ingestion has potential adverse health effects, such as an increase in methemoglobin concentration, changes in gastric mucus secretion, or genotoxic induction [1–3]. Trends in the food industry indicate an increasing consumer awareness of health and a growing preference for fewer synthetic chemical preservatives. In this pursuit, EOs, plant-derived compounds widely classified as Generally Recognized as Safe by the FDA [4], have shown significant potential due to their biological activities.

EOs derived from plants and spices are abundant in bioactive compounds, including terpenes. Ci essential oil, obtained from *Cymbopogon winterianus* – a type of citronella grass cultivated in tropical and subtropical regions of Asia, Africa, and the Americas, contains major constituents such as citronellal, geraniol, and citronellol [5]. Lc essential oil, extracted from *Litsea citrata*, a plant commonly found in southeastern Asia (e.g., China, Nepal, Japan, Taiwan, and South Korea), is primarily composed of monoterpenoids (> 90%), monoterpene hydrocarbons (up to 10%), and sesquiterpenes (up to 3%), with citral as the dominant active compound [6]. Ca essential oil, derived from *Coleus amboinicus* (also known as *Plectranthus amboinicus*), is rich in monoterpenoids (55.4%) and monoterpenes (35.3%), and sesquiterpenoids (9.3%) with carvacrol as its main bioactive component. Meanwhile, Kl essential oil, extracted from *Citrus hystrix*, a tropical fruit native to Southeast Asia, consists of monoterpene hydrocarbons (50.1%), monoterpenoids (48.6%), and sesquiterpene hydrocarbons (0.65%) with D-limonene as the primary bioactive compound. These EOs exhibit varying degrees of antimicrobial and antioxidant activities, largely attributed to the presence of monoterpenes and terpenoids such as citral, carvacrol, citronellal, and D-limonene [5–8]. While their efficacy may differ depending on the composition and concentration, the synergistic interactions among major and minor components play a crucial role in enhancing their biological properties against a broad spectrum of bacteria and fungi.

EOs are promising candidates for applications in the food industry due to their biological activities. However, the direct application of EOs in food systems may adversely affect the sensory qualities and reduce consumer acceptance of the final product. Furthermore, the poor water solubility, oxidation sensitivity, and volatility of EOs pose significant challenges. To overcome these limitations and simultaneously enhance both the efficacy and stability of EOs, encapsulation techniques have garnered significant interest [9]. A study by Y. Ruiz-Navajas et al. [4] demonstrated that incorporating *Thymus piperella* and *Thymus moroderi* EOs into chitosan edible films resulted in active films with excellent in vitro antibacterial and antioxidant activities. For meat products, Chaleshtori et al. [10] evaluated the antimicrobial activity of chitosan incorporated with lemon and oregano EOs on MC during 9 days of refrigerated storage. They reported a significant reduction in the populations of lactic acid bacteria (LAB), mold, and yeast in treated samples compared to the control. Nevertheless, previous studies have not comprehensively examined the influence of emulsification methods on the preservation efficacy of meat, nor have they characterized the antimicrobial and antioxidant properties of Ci, Lc, Ca, and Kl.

In this work, *E. coli* was chosen as a representative Gram-negative foodborne pathogen, presented in many international food standards such as Codex Alimentarius or ISO standards, while *L. plantarum* served as a representative for Gram-positive lactic acid bacteria, a group commonly associated with meat spoilage [11]. Therefore, the antimicrobial and antioxidant properties of Ci, Lc, Ca, Kl, and their mixtures were investigated. To comprehensively characterize our materials and their effects, the GC-MS method was used to determine the chemical constituents of each EOs. The antimicrobial properties of both individual compounds and their mixtures were assessed through MIC assays, along with antioxidant activity through 2,2-diphenyl-1-picrylhydrazyl (DPPH), 2,2′-azino-bis(3-ethylbenzothiazoline-6-sulfonic acid) diammonium salt (ABTS), ferric reducing antioxidant power (FRAP), and free oxygen radical assays. The FICI was determined to investigate the potential synergistic effects of the essential oil mixtures (EOMs) further. The nanoemulsions were characterized in terms of particle dimensions, surface charge (zeta potential), and their ability to combat oxidative stress and inhibit microbial growth. The nanoemulsions were incorporated into chitosan-based films, which were applied to preserve minced chicken meat for 10 days. The quality parameters assessed included weight loss, pH levels, total viable count, color values (L^*^, a^*^, b^*^), thiobarbituric acid reactive substances (TBARS), total volatile base nitrogen (TVB-N) values, and texture profile analysis (TPA).

## 2 Materials and methods

### 2.1 Materials and chemicals

Essential oils of Ci, Lc, Ca, and Kl were purchased from RUMILAB Co., Ltd., Vietnam, and were analyzed by GC–MS before use to determine their chemical profiles. Coconut oil was obtained from Luong Quoi Coconut Co., Ltd., Vietnam, while Tween 80, dimethyl sulfoxide (DMSO), ammonium chloride, and ferrous sulfate were sourced from GHTECH Co., Ltd., China. Chitosan and acetic acid were supplied by Shanghai Zhanyun Chemical Co., Ltd., China, and Duc Giang Chemicals Group JSC., Vietnam, respectively. LB broth and Nessler’s reagent were provided by HiMedia Laboratories Pvt. Ltd., India, agar from Shanghai Zhanyun Chemical Co., Ltd., China, and penicillin from Duchefa Biochemie, The Netherlands. Other chemicals, including trichloroacetic acid (TCA), sodium hydroxide, salicylic acid solution, hydrogen peroxide, ethylenediaminetetraacetic acid (EDTA), FeCl_3_, acetate buffer, and hydrochloric acid, were obtained from Xilong Scientific Co., Ltd., China. ABTS and 2,4,6-tris(2-pyridyl)-s-triazine (TPTZ) were sourced from Cool Chemistry Technology (Beijing) Co., Ltd., China, ethanol (99.5%) from Cemaco Vietnam Co., Ltd., Vietnam, and DPPH and 2-thiobarbituric acid (TBA) from Sigma-Aldrich, Germany.

### 2.2 Bacterial preparation

Suspensions of *E. coli* or *L. plantarum* were prepared by inoculating 100 μL of each strain in 8.00 mL of Luria-Bertani (LB) broth and incubating for 24 h (model MIR-162, Japan). Turbidity was measured at 600 nm using a UV-Vis spectrophotometer (model UV 1800, Shimadzu, Japan) and LB broth as the blank sample [12]. The final concentrations of *E. coli* and *L. plantarum* were determined to be 7.40 and 7.35 log_10_ CFU/mL, respectively, as calculated from an optical density standard curve.

### 2.3 Emulsion preparation

The nanoemulsions were prepared according to the procedures described by Tubtimsri et al. [13] and Yuliani and Noveriza [14], with some modifications.

#### 2.3.1 High-energy method.

The oil phase was prepared by mixing the mixture of CiCa (3 mL), coconut oil (1 mL), and Tween 80 (12 mL). The amount of EOM used was three times the MIC of CiCa. For ten minutes, the oil phase was agitated at 500 rpm using a magnetic stirrer (model MSH380-Pro, DLAB Scientific Co. Ltd, USA). The oil phase mixture is then mixed with water (84 mL) and swirled for 15 minutes at 500 rpm. In accordance with the High-Speed Shearing Homogenization (HSH) method, samples were homogenized for 3 minutes at 12,000 rpm using a high-speed shearing homogenizer (model D500, DLAB Scientific Co., Ltd., USA). Regarding the ultrasonication method, the samples were sonicated at 25 °C with a power of 600 W for 10 minutes using an ultrasonic bath (model RS22L, RAMA VIET NAM JOINT STOCK COMPANY, Vietnam).

#### 2.3.2 Phase inversion temperature process.

The oil phase was prepared by mixing the mixture of CiCa (3 mL), coconut oil (1 mL), and Tween 80 (12 mL). The amount of EOM used was three times the MIC of CiCa. For ten minutes, a magnetic stirrer (model MSH380-Pro, DLAB Scientific Co. Ltd., USA) was used to agitate the oil phase at 500 rpm. Hot water was added to the oil phase, and its temperature was maintained at 80 °C, with stirring at 500 rpm for 30 minutes. The mixture was rapidly cooled to 4 °C in an ice bath to obtain the nanoemulsions.

### 2.4 MIC determination

Bacterial suspension (1.00 mL) was added simultaneously with 0.60 mL of EOs in 8.00 mL of LB broth. The mixture was vortexed at 3000 rpm, and absorbance at 600 nm was measured using a UV-Vis spectrophotometer. Turbidity samples were measured every 30 minutes for 3 hours. EOs and their mixture were dissolved using DMSO to obtain concentrations of 2–35 μL/mL for *E. coli* and 2–40 μL/mL for *L. plantarum*. To assess synergistic effects, mixtures listed in Table 1 were prepared in specific volume ratios using DMSO. Positive and negative controls were included to validate the assay. Penicillin served as the positive control, while LB broth was used as a negative control [15,16]. The intramixture interactions among essential oils were characterized by FICI – the ratio between the MIC of the EOs mixture and the average of the MICs of the individual EOs, expressed by the following equation (1):

**Table 1:** List of 11 mixtures from citronella, litsea cubeba, coleus amboinicius, and kaffir lime essential oils.

No.	EOs and their mixtures	EOs presented	Volume ratio
1	Ci	citronella	1
2	Lc	litsea cubeba	1
3	Ca	coleus amboinicius	1
4	Kl	kaffir lime	1
5	CiLc	citronella: litsea cubeba	1:1
6	CiCa	citronella: coleus amboinicius	1:1
7	CiKl	citronella: kaffir lime	1:1
8	LcCa	litsea cubeba: coleus amboinicius	1:1
9	LcKl	litsea cubeba: kaffir lime	1:1
10	CaKl	coleus amboinicius: kaffir lime	1:1
11	CiLcCa	citronella: litsea cubeba: coleus amboinicius	1:1:1
12	CiLcKl	citronella: litsea cubeba: kaffir lime	1:1:1
13	CiCaKl	citronella: coleus amboinicius: kaffir lime	1:1:1
14	LcCaKl	litsea cubeba: coleus amboinicius: kaffir lime	1:1:1
15	CiLcCaKl	citronella: litsea cubeba: coleus amboinicius: kaffir lime	1:1:1:1


$$FICI=\frac{{MIC}_{j}}{\frac{\text{1}}{n}\times \sum_{i=\text{1}}^{n}{MIC}_{i}}\;\;\;$$ (1)

In this case, FICI is the fractional inhibitory concentration index of an EO mixture; MIC_i_ is the MIC of an individual EO; and MIC_j_ is the MIC of an EO mixture. In this study, j represents the number of each mixture in Table 1; i = 1–4 represents Ci, Lc, Ca, and Kl, respectively. An FICI ≤ 0.5 indicates synergy; 0.5 < FICI ≤ 1, additivity; 1 < FICI ≤ 4, indifference; and FICI > 4, antagonism [17].

### 2.5 Minced chicken preparation

Chitosan was dissolved in an acetic acid buffer (pH 4.5) solution to obtain a final concentration of 1%. The mixture was incorporated with the nanoemulsions to acquire an Essential oil-loaded chitosan film (ECF) and stirred at 500 rpm for 10 minutes.

For sample preparation, the meat was washed, drained, and minced, then shaped into 3 × 3 × 2 cm portions (MC). The MC blocks were coated in ECF and stored at 4 °C for 10 days. The quality criteria, including weight loss, pH, total plate count, volatile nitrogen determination, lipid oxidation, texture profile analysis, and color determination, were used to compare the preservation effectiveness of MC from three different nanoemulsion preparations.

#### 2.5.1 Weight loss.

Weight loss was determined by the difference in mass. The initial weight is denoted as W_0_, and subsequent weights W_1_, which were recorded each day. Percentage weight loss was determined using equation (2):


$$Weight\;loss\;(\%)=\frac{W_{\text{0}}-W_{\text{1}}}{W_{\text{0}}}\times \text{100}\%$$ (2)

#### 2.5.2 pH analysis.

For each measurement, 2 g of minced meat was manually mixed with 20 mL of deionized water for 5 minutes. The pH of the samples was determined using a pH meter (model MI151-US, Milwaukee Instruments, Inc., USA).

#### 2.5.3 Total plate count.

The total viable count (TVC) of the MC samples was determined using a modified procedure based on the method of [18]. Two grams of minced meat were mixed with 20 mL of deionized water and subsequently filtered. An aliquot of 100 μL from the filtrate was then plated onto plate count agar and incubated at 37 °C for 24 hours using an incubator (model MIR-162, SANYO Electric Co., Ltd., Japan). Results were reported as log_10_ colony-forming units per mL (CFU/mL).

#### 2.5.4 Volatile nitrogen determination.

The TVB-N content was determined using a slightly modified procedure by [19]. Ten grams of minced meat were mixed with 100 mL of TCA solution (20 g/L), and the mixture was incubated at room temperature for 30 minutes. The mixture was vacuum filtered. Next, the pH of the filtrate was adjusted to 6 using a NaOH solution (1 M). The mixture was transferred to a 250 mL volumetric flask and brought to volume with distilled water. The resulting extract (0.25 mL) was reacted with 0.25 mL of Nessler reagent, followed by the addition of 6 mL of distilled water (0.01% polyvinyl alcohol). It was incubated in the dark for 15 minutes before its absorbance was measured by a UV-Vis spectrophotometer at a wavelength of 400 nm. A calibration curve was created using NH_4_Cl, and the TVB-N in the sample was expressed in milligrams per gram (mg/g). The TVB-N content was calculated using equation (3):


$$X=A\times \frac{V}{m}\times D$$ (3)

Where:

**X**: the TVB-N content in the MC sample (mg/g),

**A**: the mass of nitrogen determined from the NH_4_Cl calibration curve (mg)

**V**: total sample volume (L),

**m**: the mass of MC used for analysis,

**D**: dilution factor.

#### 2.5.5 Lipid oxidation determination.

Lipid oxidation was assessed by measuring TBARS, following the method described with slight modifications by [20]. Briefly, 5 g of minced meat was homogenized with 25 mL of cold 7.5% TCA containing 0.1% EDTA and incubated for 30 minutes, followed by filtration. A 5 mL portion of the filtrate was mixed with an equal volume of freshly prepared chilled 0.02 mol/L TBA in a test tube. The mixture was then heated at 100 °C for 40 minutes. After cooling, the sample was centrifuged at 1600 rpm for 5 minutes using a centrifuge (model DM0506, DLAB Scientific Co., Ltd, USA). The absorbance of the samples was measured at 532 nm. A blank was prepared using 5 mL of TBA and 5 mL of TCA. TBARS values were expressed as milligrams of Malonaldehyde (MDA) per kilogram of meat (mg MDA/kg), calculated using equation (4):


$$TBARS\;\left(\frac{mg}{kg}\right)=\frac{A_{\text{532}}}{\text{155}}\times \frac{\text{1}}{m}\times \text{72}.\text{6}\times \text{1000}$$ (4)

Where:

**A**_**532**_: the absorbance at 532 nm,

**155**: the molar absorbance coefficient of malondialdehyde,

**m**: the mass of MC used for analysis (g),

**1**: the optical range 1 (cm),

**72.6:** The relative molecular mass of malondialdehyde.

#### 2.5.6 TPA analysis.

The texture of the chilled meat samples was evaluated using a modified procedure based on [21]. A TA-XT Plus texture analyzer (Stable Micro Systems, UK) fitted with a cylindrical aluminum probe (P/36R) was operated in TPA mode. Each sample was compressed to 50% of its original height, with a pre-test speed of 2.0 mm/s. The compression was performed twice, with a 5.0-second interval between cycles. The test speed was maintained at 1.0 mm/s, and the post-test speed was 5.0 mm/s. A trigger force of 5 g was used to initiate the measurement.

#### 2.5.7 Color determination.

The color characteristics of the samples were determined following the method described by Cheng et al. [22], using a CR-400 colorimeter (Minolta Co., Ltd., Shanghai, China). Color measurements were conducted daily, starting from the day after film coating (D0) until the 10th day of preservation. The color values were reported using the CIE LAB system, where L^*^ represents lightness, a^*^ denotes redness, and b^*^ indicates yellowness. The device was set with an 8 mm diameter measurement aperture, a 0° standard observer, and D65 illuminant, and was calibrated using a white reference tile prior to use (L* = 87.20; a* = 0.3160; b* = 0.3235). L^*^_0_, a^*^_0_, b^*^_0_ were measured on the initial day. The color measurement was expressed through equation (5):


$$\Delta E=\sqrt{{\left(L^{*}-L_{\text{0}}^{*}\right)}^{\text{2}}+{\left(a^{*}-a_{\text{0}}^{*}\right)}^{\text{2}}+{\left(b^{*}-b_{\text{0}}^{*}\right)}^{\text{2}}}$$ (5)

### 2.6 Characterization of emulsion

The technique outlined by F. Varenne et al. and Andréa A. M. Shimojo et al. was used to calculate the average particle diameter and zeta potential of the formulated nanoemulsions, which were characterized to assess their physicochemical properties [23,24]. A dynamic light scattering equipment (model SZ-100, Horiba, Japan) with a 633 nm laser, a 90° backscattering angle, and a temperature of 22 °C was used for the measurements. To stabilize the measurement temperature, samples were subjected to a tenfold dilution using ultrapure water and allowed to equilibrate for 300 seconds. Three consecutive readings were obtained after 1–1.5 mL of the diluted sample was added to a measuring cell for each measurement. The intensity-weighted size distribution was used to determine the hydrodynamic diameter. An electric field was applied to the sample to measure the zeta potential. Laser Doppler velocimetry was used to record the electrophoretic mobility of the charged particles, and the Helmholtz–Smoluchowski equation was employed to calculate the zeta potential.

### 2.7 Antioxidant assay

#### 2.7.1 ABTS assay.

The ABTS radical scavenging capacity was determined using the method described by Rumpf, Jessica et al., with minor adjustments [25]. A total of 25.0 mL of ABTS solution (7.40 mM) was prepared and mixed with 25.0 mL of potassium persulfate solution (2.45 mM) to generate ABTS ⁺ . The combined solution was maintained under dark conditions at ambient temperature for 18 hours of incubation. The resulting ABTS⁺ solution was adjusted with absolute alcohol until its absorbance reached 1.00 at 734 nm, using absolute alcohol as the blank. EOs were diluted in DMSO before use. For the assay, 0.10 mL of the diluted EOs was mixed with 3.90 mL of the adjusted ABTS⁺ solution. The mixture was kept in the dark for 20 minutes, after which the absorbance was measured at 734 nm using a UV-Vis spectrophotometer. The ABTS radical scavenging activity of the EOs was quantified by plotting the results against a standard curve prepared with trolox. The ABTS antioxidant capacity was expressed as micrograms of trolox equivalents per milliliter (µg TE/mL).

#### 2.7.2 DPPH assay.

The DPPH radical scavenging activity was evaluated according to the method outlined by Takasuka et al., with appropriate modifications. [25]. DPPH was weighed and dissolved in absolute alcohol to obtain a solution with an absorbance of 1.10 at 515 nm. Absolute alcohol was used as the blank. EOs were diluted in DMSO before use. For each test, 0.50 mL of the diluted EOs was mixed with 3.50 mL of the prepared DPPH solution. The mixture was incubated in the dark for 30 minutes, after which the absorbance was measured at 515 nm using a UV–Vis spectrophotometer. The DPPH radical scavenging activity of the EOs was quantified by plotting the results against a standard curve prepared with trolox. The DPPH antioxidant capacity was expressed as micrograms of trolox equivalents per milliliter (µg TE/mL).

#### 2.7.3 Hydroxyl radical scavenging assay.

Determination of hydroxyl radical concentration by Neil J.Stokes [26]. Preparation of reagent solutions: Ferrous sulfate (FeSO_4_) solution (9 mM), salicylic acid solution (6 mM), hydrogen peroxide (H_2_O_2_) solution (8.8 mM). The three solutions were mixed in a volume ratio of 2:5:5 (FeSO_4_: salicylic acid: H_2_O_2_) to generate the working reagent. The resulting mixture was adjusted with distilled water until an absorbance of approximately 1.10 was reached at 510 nm. Distilled water was used as the blank. EOs were pre-diluted in DMSO using a 1% Tween 80 solution as the emulsifier. For the assay, 1.6 mL of the diluted EOs was mixed with 2.4 mL of the adjusted working reagent. The mixture was incubated in the dark for 15 minutes, and the absorbance was measured at 510 nm using a UV–Vis spectrophotometer. The hydroxyl radical scavenging activity of the EOs was quantified by plotting the results against a standard curve prepared with trolox. The hydroxyl radical scavenging capacity was expressed as micrograms of trolox equivalents per milliliter (µg TE/mL).

#### 2.7.4 FRAP assay.

The FRAP assay was developed by Yang Lina et al. [27]. Preparation of reagent solutions: An acetate buffer solution (pH 3.6) was prepared and used as the base medium. A FeCl3 solution (20 mM) was prepared in distilled water, and a TPTZ solution was prepared by diluting 0.7 mL of concentrated HCl (36%) to 100.0 mL with distilled water. Then, 0.156 g of TPTZ was dissolved in 50.0 mL of the freshly prepared HCl solution. These three solutions were mixed in a 10:1:1 volume ratio (acetate buffer: TPTZ: FeCl_3_) to form the working FRAP reagent. DMSO was used as the blank. EOs were diluted in DMSO before testing. For the assay, 0.10 mL of the diluted EOs was mixed with 3.90 mL of the freshly prepared FRAP reagent. The mixture was incubated in the dark for 20 minutes, and the absorbance was measured at 593 nm using a UV–Vis spectrophotometer. The FRAP radical scavenging activity of the EOs was quantified by plotting the results against a standard curve prepared with trolox. The FRAP radical scavenging capacity was expressed as micrograms of trolox equivalents per milliliter (µg TE/mL).

### 2.8 Gas chromatography/Mass spectrometry

The chemical compounds of each essential oil were analyzed by GC (model 1980B, Agilent Technologies, USA), based on the method of Sierra et al., with minor modifications [28]. The initial oven temperature was maintained at 45 °C, followed by a gradual increase of 8 °C per minute until a final temperature of 300 °C was reached. All essential oil samples were diluted at a 1:100 ratio in diethyl ether, and 1.0 μL of each diluted sample was automatically injected using an autosampler (model 7697A, Agilent Technologies, USA). A CPWAX 52 fused silica column (50 m × 0.25 mm; 0.25 μm film thickness) was employed, with helium as the carrier gas at a flow rate of 22.1 mL/min. Mass spectrometry analysis was performed using electron spray ionization (ESI) in positive ion mode, with a mass range of 35–500 m/z scanned. The instrument parameters were set as follows: capillary voltage, 4 kV; drying gas flow, 8 L/min; nebulizing gas pressure, 40 psi; and source temperature, 250 °C. The molecular ions [M + H]+/[M + Na]+ were selected as precursor ions for compound identification. Collision energy was fixed at 70 eV, and helium served as the collision gas. Data processing and compound identification were performed using Mass Hunter software, with spectral comparisons made against the NIST14s and WILEY7 mass spectral libraries.

### 2.9 Statistical analysis

Statistical analysis was performed using Minitab software (version 19.1, Minitab Inc., Pennsylvania) with Analysis of Variance (ANOVA) at a significance level of α = 0.05. All experiments were performed in triplicate, and data are presented as the mean ± standard deviation (SD). Graphical representations were generated using OriginPro 2022 (OriginLab, Northampton, Massachusetts).

## 3 Results and discussion

### 3.1 The profile of essential oils

The chemical constituents of Ci, Lc, Ca, and Kl essential oils were identified using GC–MS. Their respective compound profiles are presented in Tables S1-S4 in S2 File, with the chromatographic patterns illustrated in Figs S1 A-D in S2 File Analysis of Ci essential oil revealed the presence of 61 compounds, including monoterpenes, monoterpenoids, sesquiterpenes, alcohols, ketones, and hydrocarbons. Geraniol was the predominant component (48.04%), followed by geranyl acetate (11.83%), tetracontane (9.44%), and citral (4.97%). Minor constituents included β-citronellol (2.00%), trans-β-caryophyllene (2.53%), and D-limonene (2.03%). The obtained profile corresponds to previously reported data on Cymbopogon winterianus, in which oxygenated monoterpenes were found to be predominant. Geraniol has been reported as the principal compound, ranging from 35% to 42%, followed by significant levels of geranyl acetate and citronellol, depending on the extraction methods applied, such as steam distillation and hydrodistillation [5,29].

The analysis of Lc essential oil identified a total of 73 compounds, dominated by monoterpenes and monoterpenoids. Citral was the most abundant compound, comprising 20.36%, followed by Δ3-carene (15.17%), 2,6-Octadienal, 3,7-dimethyl-, (Z)- (15.00%), D-limonene (6.82%), and geraniol (3.54%). Several oxygenated monoterpenes were also identified, including linalool (0.57%), eucalyptol (1.59%), and isocineole (1.93%). The essential oil also contained sesquiterpenes, including longifolene (2.31%), trans-β-caryophyllene (1.10%), and humulene (0.17%). Some minor components are α-pinene (0.09%), trans-isoeugenol (0.08%), and β-citronellol (0.03%). These data were consistent in overall chemical classes with those reported by Yang et al., where geranial (27.5%), neral (23.6%), and D-limonene (18.8%) were the dominant compounds in fruit-derived essential oil [30]. In Vietnamese samples, Hung et al. geranial (32.0%), neral (24.3%), citronellal (14.2%), and limonene (10.1%) as key constituents [31]. However, the citral content in this study was considerably lower, and Δ3-carene appeared in a markedly higher proportion than in previously published data.

A total of 53 chemical constituents were detected in the Ca essential oil. It was rich in oxygenated monoterpenes, with isobornyl acetate (21.17%) as the principal component, followed by 1,8-cineole (10.36%), trans-β-caryophyllene (8.25%), 3-Cyclohexen-1-ol, 4-methyl-1-(1-methylethyl)-, (R)- (12.04%), and (+)-2-bornanone (3.99%). Linalool (6.98%), D-limonene (2.24%), and nerol (1.54%) were also found in considerable amounts. Minor compounds like thymol (0.83%), eugenol (0.23%), and caryophyllene oxide (0.19%) were present as well. These results differ in proportions when compared to other research. In D. T. B. Hanh et al. reported oils from Dak Lak to be dominated by 2,3,5,6-tetramethylphenol (67.94%), caryophyllene (9.74%), trans-α-bergamotene (5.82%), α-humulene (3.20%), γ-terpinene (2.5%), o-cymene (1.90%), and terpinen-4-ol (1.14%) [32].

In Kl essential oil, there are 44 compounds; 64.15% of the total composition was D-limonene, making it the dominant component. Additional major compounds included γ-terpinene (8.31%), bicyclo [3.1.1] heptane, 6,6-dimethyl-2-methylene-, (1S)- (9.82%), citral (0.82%), and trans-α-bergamotene (0.48%). A variety of monoterpenes and sesquiterpenes, such as sabinene (1.31%), β-myrcene (1.19%), and β-bisabolene (0.82%), were detected. While linalool (0.10%) and geraniol (0.03%) were present in trace amounts, the oil also contained rare triterpenoids and alkanes, including α-amyrin (0.53%) and tetracontane (0.89%). In a separate analysis, GC–MS identified 27 components in Kl peel essential oil, with β-pinene (27.37%), β-phellandrene (22.69%), D-limonene (16.21%), and citronellal (12.43%) as the four major constituents, respectively [33]. Additional components included 3-carene (3.95%), terpinen-4-ol (2.63%), α-terpineol (2.56%), linalool (2.17%), citronellol (2.06%), and γ-terpinene (1.53%), while 17 compounds were present at trace levels below 1.5%. Previous reports have shown similar variability depending on the extraction method and geographic origin. An et al. identified β-pinene (33.94%), sabinene (22.88%), D-limonene (15.85%), and citronellal (14.79%) in Vietnamese peel oil [34].

Overall, the variation in chemical composition and biologically active constituents among Ci, Lc, Ca, and Kl, as reported by researchers, varied significantly due to factors such as genetic background, geographic origin, plant part used, and extraction method. The dominance of specific compounds in each oil reflects underlying chemotypic diversity and environmental influence.

### 3.2 Antioxidant activity of essential oils and their mixture

**Fig 1:**
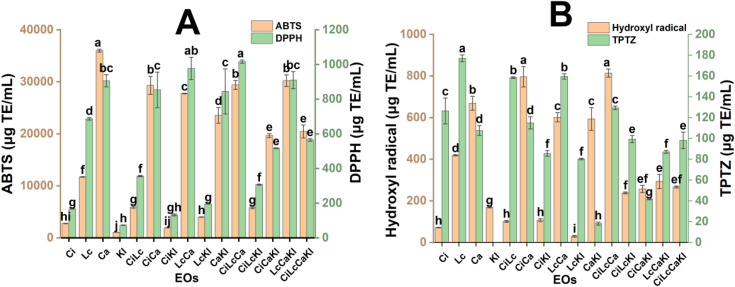
The antioxidant activities of four essential oils and their mixtures. (A) ABTS and DPPH assays; (B) Hydroxyl radical and FRAP assays. Different characters show significant statistical distinctions (p < 0.05).

Figs 1A, 1B present the antioxidant activities of individual essential oils (EOs) and their combinations, assessed through four assays: ABTS, DPPH, hydroxyl radical scavenging, and FRAP. Among the individual EOs, Ca exhibited the highest antioxidant activity in the ABTS, DPPH, and hydroxyl radical scavenging assays, with values of 35996 μg TE/mL, 906 μg TE/mL, and 669 μg TE/mL, respectively. However, Ca showed the weakest performance in the FRAP assay, of 108 μg TE/mL. Lc demonstrated the strongest ferric reducing power in the FRAP assay (177 μg TE/mL). Kl showed the lowest antioxidant activities in both ABTS and DPPH assays, with values of 1066 μg TE/mL and 73 μg TE/mL, respectively. Meanwhile, Ci recorded the lowest hydroxyl radical scavenging capacity at 71 μg TE/mL.

Ca exhibits the strongest antioxidant capacity due to the presence of isobornyl acetate, eucalyptol, and 3-cyclohexen-1-ol. These molecules contain ester and hydroxyl groups that are capable of facilitating electron and hydrogen transfer. This structural feature allows them to stabilize radical intermediates, effectively preventing the propagation of free radical reactions. This finding aligns with the results of Viviana Donoso-Bustamante et al., who reported that the presence of a methoxyl group on the phenyl ring significantly enhances antioxidant activity. [35]. These abilities allow Ca to trap the free radicals efficiently. Chia-Wen Lin et al. showed that β-caryophyllene was one of the best antioxidant compounds in the DPPH assay [36]. This substance is also present in Ca’s major components. Kl displaces low antioxidant activity due to the structural nature of its major compounds. D-limonene and γ-terpinene lack the electron-donating groups necessary for radical scavenging and possess a hydrophobic nature that prevents them from interacting effectively with hydrophilic free radicals. Ci performed poorly in antioxidant assays due to the prevalence of linear, non-conjugated compounds such as geraniol and geranyl acetate. These molecules lack the phenolic or reactive functional groups required to effectively stabilize electron systems, thereby limiting their radical scavenging capacity. Lc demonstrated potent antioxidant activity in the FRAP assay, attributed to the presence of aldehyde-containing compounds such as citral and 2,6-octadienal. These molecules possess conjugated double bonds and strong reducing capabilities, which facilitate the interaction with metal ions required for this assay. In contrast, Ca showed the weakest performance in the FRAP assay due to the absence of aldehydes in its major compounds [37,38].

Regarding EOMs, results from ABTS and FRAP assays indicated that their antioxidant activity corresponds approximately to the mean value of the individual essential oils. In the ABTS assay, CiCa, CiLcCa, and LcCaKl showed the highest antioxidant activity, with values of 29,329 μg TE/mL, 29,413 μg TE/mL, and 30,204 μg TE/mL, respectively. The lowest ABTS value was 1925 μg TE/mL for CiKl. In the FRAP assay, CiLc (158 μg TE/mL) and LcCa (160 μg TE/mL) exhibited the highest ferric reducing power. CaKl (18 μg TE/mL) showed the weakest. DPPH and hydroxyl radical scavenging assays revealed a different trend in antioxidant activity. CiLcCa was the most potent in both assays, with 1,016 μg TE/mL in DPPH and 814 μg TE/mL in hydroxyl scavenging. LcCa (977 μg TE/mL in DPPH) and CiCa (796 μg TE/mL in hydroxyl scavenging) also demonstrated high activity. The lowest activities were observed for CiKl in the DPPH assay (132 μg TE/mL) and for LcKl in the hydroxyl radical scavenging assay (30 μg TE/mL). The combination of EOs can yield a synergistic effect, significantly enhancing their overall antioxidant activity. The linear hydrophobic chains and aromatic rings can engage in stacking, leading to π-π interactions and hydrophobic associations. These interactions not only improve the dispersion and solubility of hydrophilic functional groups (e.g., hydroxyl, carbonyl, methoxyl) but also enhance their accessibility and reactivity with free radicals, thereby elevating the overall antioxidant efficacy of the blend [39,40].

### 3.3 MIC of essential oils and their mixture

Table 2, Figs S2 A1-A16, and Figs S3 B1-B16 in S2 File show the antibacterial activity of EOs and their mixtures with two bacteria: *E.coli* (gram-negative) and *L.plantarum* (gram-positive). The MIC (μL/mL) for each EOs and their mixtures was defined as the lowest concentration in which the turbidity of microbial culture remains unchanged during the experimental period. The efficacy of the essential oil (EOs) mixture in controlling the growth of representative Gram-negative (*E. coli*) and Gram-positive (*L. plantarum*) bacteria validates its potential as a broad-spectrum antimicrobial. This suggests that the mixture could be employed as a natural preservative to enhance the microbial safety and storage stability of meat. Penicillin exhibited potent antibacterial activity against *E. coli* (1 μL/mL) but was less effective against *L. plantarum* (7.5 μL/mL). Among the individual essential oils, Ci was the most effective for both strains (5 μL/mL), followed by Lc (7.5 μL/mL). Ca exhibited the weakest effect, requiring 35 μL/mL and 30 μL/mL for *E. coli* and *L. plantarum,* respectively. Kl performed well on *L. plantarum* (8 μL/mL) but showed limited impact on *E. coli* (20 μL/mL).

**Table 2:** Antibacterial activities and FICI of four essential oils and their mixtures.

No.	Samples	*L.Plantarum*		*E.Coli*	
		MIC (μL/mL)	FICI	MIC (μL/mL)	FICI
1	Ci	5		7.5	
2	Lc	7.5		10	
3	Ca	30		35	
4	Kl	8		20	
5	CiLc	6	0.96	8	0.91
6	CiCa	4	0.23	5	0.24
7	CiKl	2	0.31	8	0.58
8	LcCa	2	0.11	20	0.89
9	LcKl	2	0.26	10	0.67
10	CaKl	4	0.21	25	0.91
11	CiLcCa	4	0.28	15	0.86
12	CiLcKl	6	0.88	15	1.20
13	CiCaKl	8	0.56	20	0.96
14	LcCaKl	20	1.32	20	0.92
15	CiLcCaKl	10	0.79	20	1.10
16	Penicillin	7.5		1	

A lower FICI value indicates a more effective antibacterial activity of the EOs combination, with values ≤ 0.5 considered a synergistic effect. LcCa exhibited the most potent inhibitory effect against *L. plantarum*, with an MIC of 2 μL/mL and a FICI of 0.11. In contrast, the LcCaKl mixture demonstrated the weakest effect, with an MIC of 20 μL/mL and an FICI of 1.32. Several EO combinations exhibited synergistic effects (FICI ≤ 0.5) against *L. plantarum*, including CiCa, CiKl, LcCa, LcKl, CaKl, and CiLcCa. Their corresponding MIC values were 2 μL/mL (CiKl, LcCa, LcKl) and 4 μL/mL (CiCa, CaKl, CiLcCa). In the case of *E. coli*, both MIC and FICI values were generally higher than those observed for *L. plantarum*, with MIC values ranging from 5 to 25 μL/mL and most FICI values exceeding 0.5. CiCa was the only mixture to exhibit a synergistic effect against *E. coli*, with an FICI of 0.24 and an MIC of 5 μL/mL.

The antibacterial mechanism is driven by lipophilic terpenes such as D-limonene, trans-β-caryophyllene, isobornyl acetate, 3-cyclohexen-1-ol, 4-methyl-1-(1-methylethyl)-, (R)-, and eucalyptol. These compounds can disrupt the integrity of the cytoplasmic membrane, thereby altering the membrane potential and facilitating the leakage of essential intracellular constituents, such as ATP [41]. Moreover, Zulfiqar Ahmad et al. [42] suggested that hydroxyl groups, such as geraniol, 3-cyclohexen-1-ol, 4-methyl-1-(1-methylethyl)-, (R)-linalool, and p-menth-1-en-3,8-diol, exhibit potential to downregulate proteins, including ATPase. Hydrophobic hydrocarbon constituents present in essential oils (EOs) may impair ATPase activity by accumulating within the lipid bilayer, thereby disrupting lipid-protein interactions or through direct binding to the enzyme [25]. Terpenoid compounds like geraniol, geranyl acetate, isobornyl acetate, 3-cyclohexen-1-ol, 4-methyl-1-(1-methylethyl)-, (R)-, eucalyptol (1,8-cineole) directly affect the integrity and fluidity of the membrane structure, and integrate into the aqueous-facing polar region of the membrane structure [43]. Through hydrophobic contacts and hydrogen bonding, the compounds exhibit binding affinity toward the membrane and periplasmic domains. As a result, the reaction rate of bacteria can be decreased against antibacterial compounds outside [44]. Hydrocarbons like tetracontane, alpha-bergamotene, and 17-pentatriacontene showed lipophilic properties. The interaction of these molecules with the lipid bilayer’s apolar tails results in the formation of membrane microchannels. The formation of microchannels facilitates the release of essential intracellular components, such as proteins, reducing sugars, ATP, and DNA, which are toxic to *E. coli* and *L. plantarum* [15,45]. Di Pasqua et al. demonstrated the relationship between the alteration in membrane cells and EOs’ antibacterial activities [46]. 6-Octenal, 3,7-dimethyl-, (R)-, (+)-2-bornanone are aldehyde compounds that promote the production of saturated fatty acids, thereby reinforcing the structural rigidity of the cellular membrane by modifying the lipid profile of L. plantarum and *E. coli*. This activity causes cytoplasmic material to coagulate, decreases the respiratory rate, and leads to membrane depolarization and destruction [43]. *L. plantarum* is more vulnerable to damage than *E. coli* bacterial cells under the effect of essential oils. The characteristics of the bacterial cell membrane could be the cause of this result. *L. plantarum*’s cell wall structure consists of a single peptidoglycan layer that is not effective at preventing essential oil invasion [15]. On the other hand, *E. coli* has a complex cell wall structure that consists of lipopolysaccharides on the outer membrane, peptidoglycan layers, and membrane proteins. These layers act as a selective barrier, preventing antimicrobial agents from entering the internal bacterial cells [15]. Polly Soo Xi Yap et al. demonstrated how essential oils can disrupt membranes, and a similar finding was also reported [47]. Although CiCa was not the most effective against *L. plantarum* (MIC of 4 μL/mL and FICI of 0.23), it demonstrated the highest efficacy against *E. coli*, making it the most promising candidate for MC preservation.

### 3.4 Characteristic of nanoemulsion

#### 3.4.1 Particle size and Zeta potential.

Nanoemulsions were characterized using the Phase Inversion (PI) method with a formulation ratio of essential oil, coconut oil, and Tween 80 at 3:1:12, maintaining an oil phase concentration of 16%. HSH was implemented under equivalent formulation conditions with a stirring intensity of 12,000 rpm for a duration of 3 minutes. The ultrasonic emulsification (UE) method was applied to identical samples, subjected to sonication at 600 W for 10 minutes. Particle size plays a crucial role in determining the visual appearance, bioavailability, optical properties, and rheological behavior of nanoemulsions. In addition to size, the polydispersity index (PDI) offers insights into the uniformity of droplet distribution within the system. Zeta potential, a key indicator of surface charge in colloidal dispersions, serves as a predictive parameter for assessing the stability of colloidal dispersions.

Among the techniques, PI yielded the most favorable characteristics with the smallest particle size (14.17 nm), lowest PDI (0.09), and most negative zeta potential (−23 mV). HSH exhibited the highest Z-average (43.13 nm) and PDI (0.58), along with a more negative zeta potential (−19 mV), compared to UE. UE showed a lower Z-average (27.05 nm) and PDI (0.24), but a less harmful zeta potential (−17.33 mV), indicating an opposite trend to HSH.

Statistical analysis (ANOVA followed by Fisher’s LSD) at a significance level of p < 0.05. Different letters within a row signify a statistically significant difference between the means of the compared methods.

As shown in Table 3, differences in particle size, uniformity, and surface charge among the three methods arise from their distinct formation mechanisms. The PI method exploits thermodynamic changes in nonionic surfactants during cooling below the inversion temperature. This triggers an entropy-driven disruption of the bicontinuous structure, leading to the spontaneous formation of uniformly small droplets. As a result, the system exhibits a low Z-average, narrow PDI, and high zeta potential, reflecting a homogeneous and stable dispersion. HSH, based on mechanical shear and turbulence from rotor-stator interactions, generates insufficient energy to reduce droplets smaller than PI. It produces larger particles with broader size distribution and suboptimal zeta potential, reflecting poor uniformity and limited colloidal stability. UE relies on acoustic cavitation from high-frequency ultrasound, generating localized shear and shock waves that reduce the size of droplets. The resulting emulsions exhibit intermediate droplet sizes and moderate uniformity [48]. During the ultrasonication process, negatively charged compounds are degraded, resulting in a decrease in the absolute value of the zeta potential. Among the three, PI proved most efficient, producing the smallest, most uniform, and electrostatically stable nanoemulsions, highlighting its superiority in emulsification performance.

**Table 3:** Characteristics of Nanoemulsions from UE, HSH, and PI.

Method/Criteria	UE	HSH	PI	NE
Z-average (nm)	27.05 ± 1.36^b^	43.13 ± 9.78^a^	14.17 ± 0.40^b^	
PDI	0.24 ± 0.02^b^	0.58 ± 0.07^a^	0.09 ± 0.09^c^	
Zeta potential (mV)	−17.33 ± 1.01^a^	−19.00 ± 0.69^b^	−23.00 ± 0.60^c^	
MIC of *E.coli* (μL/mL)	4.5	4.5	4.5	5
MIC of *L.Plant* (μL/mL)	3	3	3	4
ABTS (mg TE/mL)	95.80 ± 1.59^c^	110.03 ± 6.36^b^	119.76 ± 3.66^a^	29.33 ± 1.75^d^
DPPH (mg TE/mL)	1.59 ± 0.01^b^	1.61 ± 0.04^b^	1.43 ± 0.08^a^	0.85 ± 0.1^c^
Hydroxyl radical (mg TE/mL)	7.87 ± 0.33^b^	7.92 ± 0.04^b^	8.28 ± 0.08^a^	0.80 ± 0.05^c^
FRAP (mg TE/mL)	3.10 ± 0.01^b^	3.26 ± 0.07^b^	3.65 ± 0.16^a^	0.11 ± 0.01^c^

#### 3.4.2 Biological activities of nanoemulsion.

Table 3 illustrates the antioxidant activities of the CiCa EOM and its nanoemulsions prepared using three different methods: UE, HSH, and PI. The previously determined MIC of NE was 5 μL/mL against *E. coli* and 4 μL/mL against *L. plantarum*. Emulsification using ultrasound, high shearing homogenization, and PI reduced MIC values to 4.5 μL/mL for *E. coli* and 3 μL/mL for *L. plantarum*, indicating enhanced antimicrobial effectiveness. Although all methods produced similar antibacterial effects, they differed in antioxidant-related parameters. In Table 3, antioxidant capacity was assessed via four assays: ABTS, DPPH, hydroxyl radical scavenging, and ferric reducing antioxidant power (FRAP). Among the tested methods, PI demonstrated the most antioxidant activity, as indicated by ABTS (120 mg TE/mL), DPPH (1.43 mg TE/mL), hydroxyl radical scavenging (8.28 mg TE/mL), and FRAP (3.65 mg TE/mL). In contrast, the non-emulsification CiCa showed the weakest antioxidant capacity across all assays, with values of 29 mg TE/mL (ABTS), 0.85 mg TE/mL (DPPH), 0.80 mg TE/mL (hydroxyl radical scavenging), and 0.11 mg TE/mL (FRAP). Both UE and HSH yielded relatively high and comparable antioxidant activities. The UE method resulted in 95.8 mg TE/mL (ABTS), 1.59 mg TE/mL (DPPH), 7.87 mg TE/mL (hydroxyl radical scavenging), and 3.10 mg TE/mL (FRAP). Similarly, the HSH method produced values of 110.03 mg TE/mL, 1.61 mg TE/mL, 7.92 mg TE/mL, and 3.26 mg TE/mL in the respective assays.

Lou Zaixiang et al. estimated that EO nanoemulsion was making way for the effective delivery of active ingredients. Their large surface area allowed active compounds to react quickly so that the antioxidant activities can be enhanced [49]. Additionally, the hydrocarbon chains in CiCa components exhibit strong hydrophobicity, which limits their solubility in water. When CiCa was emulsified, the alkyl chains of oleate in Tween 80 associated with the lipophilic components of CiCa, facilitating the formation of micelle structures. Sanjana Senthilkumar et al. demonstrated that micelle formation, by providing a hydrophobic core, significantly improved the aqueous solubility [50]. This enhanced solubility and dispersion in polar media facilitates greater interaction with free radicals, thereby potentially increasing the antioxidant activity of CiCa.

### 3.5 Comparison of three emulsifying methods in minced chicken preservation

#### 3.5.1 Water retention capacity.

**Fig 2:**
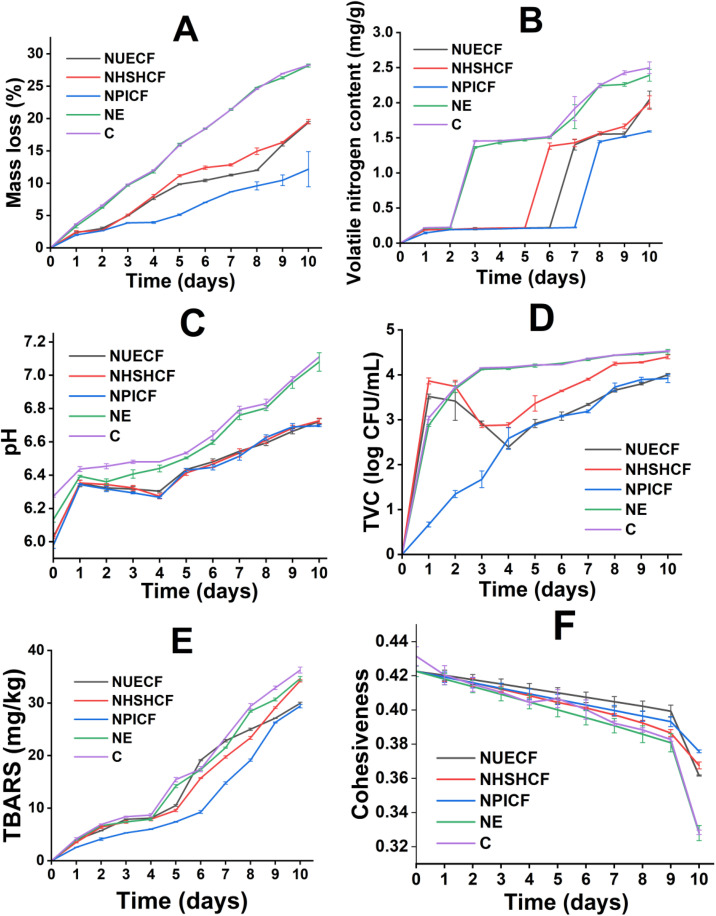
The criteria of MC treated by C, NE, NUECF, NHSHCF, NPICF, and C for 10 days. (A): The variation in mass of MC; (B): The variation in volatile nitrogen content of MC; (C): The variation in pH of MC; (D): The variation in TVC of MC; (E): The variation in TBARS of MC; (F): Variation in Cohesiveness.

Fig 2A illustrate the variation of chick meat weight for 10-day preservation using control (C), non-emulsified essential oil (NE), nanoemulsion ultrasonic emulsification Chitosan Film (NUECF), Nanoemulsion High-speed Shearing Homogenization Chitosan Film (NHSHCF), and NPICF treatments. On the first day, the MC weight reduced by 3.69%, 3.35%, 2.38%, 2.46%, and 2.00% under C, NE, NUECF, NHSHCF, and NPICF treatments, respectively. On the 10th day, the weight of MC decreased to 28.26%, 28.19%, 19.38%, 19.52%, and 12.16% under C, NE, NUECF, NHSHCF, and NPICF treatments, respectively. The highest weight loss of the NE treatment is attributed to the absence of the chitosan film, which acts as a barrier to prevent moisture evaporation [51]. The lack of a barrier creates significant differences in moisture between MC and the external environment, facilitating water diffusion. NPICF treatments exhibited the lowest weight loss among the three preservation methods. The nanoemulsion from the PI method has the smallest particle sizes, which creates a more uniform distribution of EO [52]. This uniform distribution provides a high density of EO in NPICF, which creates a uniform hydrophobic environment [53]. This environment limits water evaporation from the internal environment to the external environment compared to the nanoemulsion system with larger particles. Moreover, the moisture loss was prevented by the high surface tension property of chitin presented in ECF. Instead et al. reported a 40.5% weight loss in unpackaged samples, while those packaged with gelatin and chili seed oil nanoemulsion pouches (61–127 nm) showed reduced losses of 16.2–20.4% [54].

#### 3.5.2 Total volatile nitrogen variation.

TVB-N is a widely used indicator of meat spoilage, as it reflects the accumulation of volatile nitrogenous compounds during storage [19,55]. According to the Vietnamese standard TCVN 12429–3:2021, the TVB-N levels in chilled chicken meat must not exceed 20 mg/100 g. This value serves as a threshold rather than a fixed storage time since it depends on industrial practices. Fig 2B and Table S5 in S2 File illustrate the changes in TVB-N levels over time. On the 1st day, TVB-N contents in the MC were 0.22, 0.21, 0.19, 0.19, and 0.15 mg/100g under C, NE, NUECF, NHSHCF, and NPICF treatments, respectively. After 10 days of storage, TVB-N contents of MC treated by C, NE, NUECF, NHSHCF, and NPICF increased to 2.50, 2.39, 2.05, 2.00, and 1.59 mg/100g, respectively. The most significant increase in TVB-N contents of MC treated by C and NE can be attributed to the rapid proliferation of spoilage bacteria. These bacteria’s activity can degrade meat protein into ammonia and other nitrogenous compounds [56]. However, nanoemulsions from PI have high antibacterial activity and permeability, which can inhibit the activity of spoilage bacteria. This inhibition reduces the deterioration of meat caused by spoilage bacteria, thereby decreasing the accumulation of biogenic amines and prolonging the preservation time of the meat. Anvar et al. reported that minced meat samples coated with a film containing nano-encapsulated essential oil from Anethum graveolens L. seeds (particle size 141–165 nm) exhibited lower TVB-N levels (27 mg N/100 g) compared to the control (33.56 mg N/100 g) after 18 days [57].

#### 3.5.3 pH variation.

The pH level serves as a key indicator of the freshness of poultry meat [58]. The pH values are shown in Fig 2C. The pH values of the MC under C, NE, NUECF, NHSHCF, and NPICF treatments on the first day of storage were 6.44, 6.39, 6.35, 6.35, and 6.34, respectively. Then, NE samples exhibited the lowest pH on the 2nd day, whereas MC preserved by NUECF, NHSHCF, and NPICF showed the lowest pH on the 4th day. After 10 days, pH values increased to 7.11, 7.08, 6.72, 6.73, and 6.7 for the C, NE, NUECF, NHSHCF, and NPICF treatments, respectively. After storage time, pH differed between C and NE with the ECF-coated samples, whereas there was no significant variation among the three preservation methods. The decrease in pH was linked to the breakdown of glycogen, ATP, and creatine phosphate. The degradation of these nutrients was caused by the activity of aerobic microorganisms, which produced lactic acid [59,60]. After prolonged storage time, microbial and enzymatic activity degrade protein. This activity forms nitrogenous compounds, such as ammonia and amines, increasing the pH levels [61,62]. Terpenoids such as geraniol, citral, and thymol in CiCa incorporated into the ECF mitigate pH fluctuations and delay meat spoilage. The small particle sizes of PI-based nanoemulsion facilitate the diffusion of bioactive compounds into the cell membranes of the spoilage and lactic acid bacteria. This action inhibits lactic fermentation and the chemical decomposition of meat. Lin et al. reported minimal pH change in pork preserved with β-glucan films containing 5% lemon essential oil nanoemulsions, attributing it to the film’s barrier effect against moisture and oxygen [58].

#### 3.5.4 Total viable count.

Microorganisms contribute to protein spoilage, so TVC is commonly used to assess microbial contamination [63]. Following the Vietnamese standard TCVN 12429–3:2021, the acceptable viable count for chilled chicken meat must be below 5,000,000 CFU/g (equivalent to 6.70 log_10_ CFU/g). Fig 2D show TVC changes in MC samples during storage. The CFU/g values were converted from the CFU/mL data in Fig 2D based on the sample homogenization factor of 10 used in the analysis. On the first day, the TVC values of MC preserved by C, NE, NUECF, NHSHCF, and NPICF treatments were 3.04 log_10_ CFU/g, 2.87 log_10_ CFU/g, 3.52 log_10_ CFU/g, 3.86 log_10_ CFU/g, and 0.67 log_10_ CFU/g, respectively. After 10 days of the storage process, the TVC values of MC preserved by C, NE, NUECF, NHSHCF, and NPICF increased to 4.53 log_10_ CFU/g, 4.51 log_10_ CFU/g, 4.00 log_10_ CFU/g, 4.40 log_10_ CFU/g, and 3.92 log_10_ CFU/g, respectively. During storage time, the decomposition of meat is influenced by two groups of microorganisms: lactic acid bacteria and aerobic bacteria. The proliferation of microbes in MC is facilitated in the absence of ECF, which acts as a barrier to prevent the attachment of external microorganisms. Additionally, the lowest TVC values of MC treated by NPICF result from the most significant improvement of EO’s antibacterial activity. Nanoemulsion from PI has the smallest particle size, which improves the penetration of EO into bacterial membranes. This penetration disrupts membrane integrity, causing the leakage of cellular contents [64]. Zhang et al. and Ma et al. reported that MC treated with Moringa oleifera seed oil nanoemulsions with the ultrasonication method (17.840 nm) exhibited significantly lower TVC, 6.39 log_10_ CFU/g, during refrigerated storage, compared to the control samples with TVC of 7.57 log_10_ CFU/g [65,66].

#### 3.5.5 Lipid oxidation.

Lipid oxidation is commonly assessed by measuring MDA through the TBARS assay, with higher values indicating greater peroxidation [67]. Fig 2E show the variation of TBARS value throughout preservation. The initial TBARS values of the MC were 4.18, 3.93, 3.97, 3.5, and 2.56 mg/kg under C, NE, NUECF, NHSHCF, and NPICF treatments, respectively. The TBARS values of MC treated with C, NE, NUECF, NHSHCF, and NPICF increased to 36.25, 34.64, 29.95, 34.22, and 29.4 mg/kg, respectively, after 10-day preservation. This most significant increase in TBARS values of MC treated by NE can be attributed to the auto-oxidation of lipids under the influence of the external environment [68]. The lowest TBARS values of MC treated by NPICF result from the creation of the oxygen barrier on the meat surface. This surface acts as a thin buffer layer to prevent the permeation of oxygen from the external environment into the internal MC [69]. This action helps reduce the auto-oxidation of lipids. Additionally, the small particle sizes of PI-based nanoemulsions significantly improve the antioxidant capacity of EOs in an aqueous environment. That property of nanoemulsion facilitates the movement of EOs into the MC interior, which assists in reducing the auto-oxidation of lipids [70]. Cheng et al. reported that MC samples coated with a Pickering emulsion film stabilized by bacterial nanocellulose (particle size 1.75–2.25 μm). This system exhibited lower TBARS values (0.47 mg/kg) compared to the control (1.00 mg/kg) after 9 days of storage [71].

#### 3.5.6 Effect on the color of minced chicken meat.

**Fig 3:**
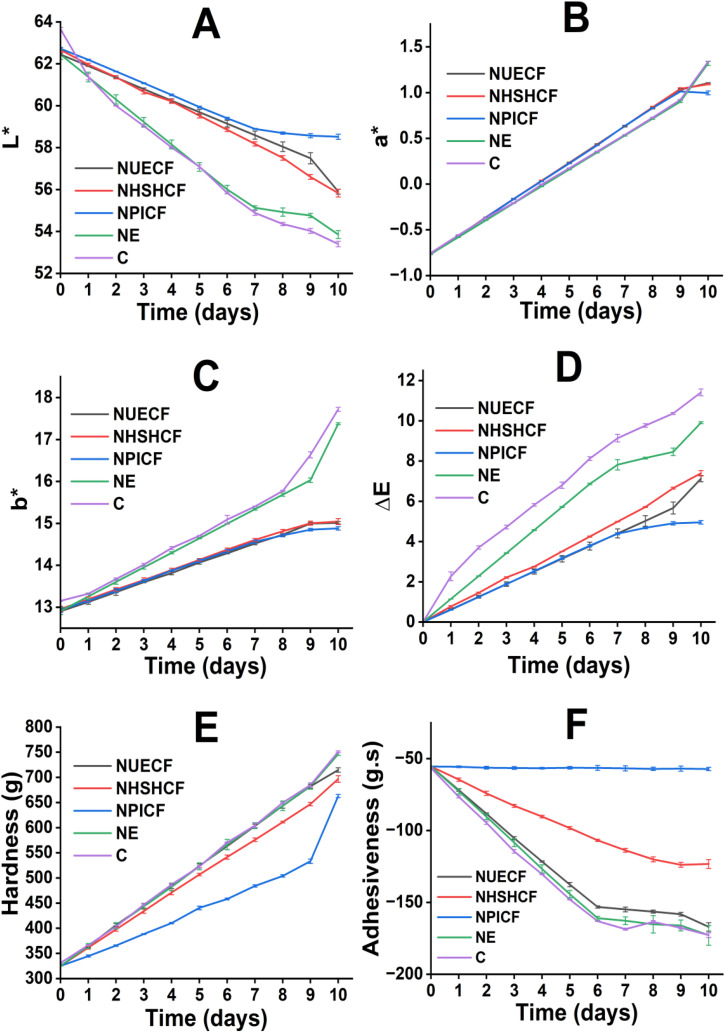
The color changes and TPA values of MC were treated by NE, NUECF, NHSHCF, NPICF, and C over 10 days. (A): Variation in L* value (lightness); (B): Variation in a* value (redness); (C): Variation in b* value (yellowness); (D): Variation in total color difference (ΔE); (E): Variation in Hardness; (F): Variation in Adhesiveness.

Color is a key sensory attribute that influences consumer acceptance and serves as a direct indicator of meat quality [63,72]. Figs 3A-D show the color changes of the MC samples during storage. After 10 days of storage, MC treated by C and NE showed a significant decrease in lightness (L*), followed by NUECF and NHSHCF treatment, while MC treated by NPICF experienced a slight reduction. The b* (yellowness) and a* (redness) values of MC treated by C and NE were significantly reduced, whereas those of NPICF were only slightly decreased. On the 1st day, the ΔE value of the MC treated by C, NE, NUECF, NHSHCF, and NPICF was 2.3, 1.1, 0.6, 0.8, and 0.6, respectively. After 10 days, the ΔE value of MC treated by C, NE, NUECF, NHSHCF, and NPICF increased to 11.4, 9.9, 7.1, 7.4, and 5, respectively. The nanoemulsion from PI, UE, and HSH contains eucalyptol and isobornyl acetate, which chelate with oxidation agents. This action reduces the conversion of bright red oxymyoglobin and deoxymyoglobin into metmyoglobin, limiting an increase in the redness and yellowness of meat [73–75]. Additionally, the smallest particle sizes of the nanoemulsion from PI facilitate the reaction between bioactive compounds and oxidation agents, increasing the effectiveness of bioactive compounds in meat preservation [76]. This trend was consistent with Chen et al., who employed EO-loaded chitosan-gum arabic to enhance the conservation of meat [77]. Moreover, the effect on the color of minced chicken meat is illustrated in Fig S4 in S2 File, which shows the actual appearance of the minced chicken cubes subjected to different treatments over a 10-day storage period.

#### 3.5.7 Effect on the TPA of meat.

Texture is a key quality attribute of MC [78]. Fig 2F, Figs 3E-F show the TPA values of MC during the preservation period. Initially, the hardness of the MC samples was 325 g, adhesiveness was −55.4 g.s, and cohesiveness was 0.423. After 10 days of preservation, there was a significant increase in the hardness of MC treated by C and NE, while the hardness of MC treated by NPICF witnessed a slight rise. The adhesiveness and cohesiveness of MC treated by C and NE experienced a substantial decrease, while that of NPICF was slightly reduced. During storage time, PI-based nanoemulsions limit the oxidation of collagen in MC by free radicals, thereby reducing the formation of cross-links among collagen molecules. The appearance of cross-linking generates dense networks of degenerated collagen in MC [79]. Additionally, PI-based nanoemulsion can prevent water evaporation from MC to the external environment [80]. The combination of two activities results in a significant increase in the MC hardness treated by NPICF compared to C, NE, NUECF, and NHSHCF. Regarding adhesiveness and cohesiveness, the high antibacterial activity of PI-based nanoemulsion reduces the protein hydrolysis of MC by spoilage bacteria. This activity maintains protein-protein interaction and limits the generation of products from protein hydrolysis. The adhesiveness and cohesiveness of MC remain during the preservation period [81]. Sayadi et al., Xiong et al., and Rather et al. reported a significant increase in hardness in unpackaged samples during storage, while those packaged with gelatin and chili seed oil nanoemulsion pouches (61–127 nm) exhibited better texture during the preservation process [82]. Therefore, NPICF is a suitable method for extending the preservation of MC among the three approaches.

Building upon these findings, Chaleshtori et al. incorporated lemon and oregano EO at concentrations of 0.5%, 1%, and 2% (w/v) into 2% chitosan coating and applied it to broiler breast meat using a dipping method [10]. The EO showed strong antimicrobial activity (MIC 1.41–11.25 mg/mL for lemon oil; 2.81–22.5 mg/mL for oregano oil). Compared to the control, the coatings significantly reduced total mesophilic bacteria until day six, while inhibiting lactic acid bacteria, Enterobacteriaceae, yeasts, and molds throughout nine days of refrigerated storage. Notably, 1% lemon oil yielded the best sensory acceptance (p < 0.01). Similarly, Guerra et al. evaluated chitosan coatings (4 and 8 mg/mL) combined with *Mentha piperita* or *Mentha villosa* EO (1.25–5 μL/mL) for postharvest control on table grapes [83]. The coatings of chitosan 4 mg/L and *Mentha piperita* or *Mentha villosa* EO at 2.5 and 5 µL/mL effectively delayed growth and reduced infections caused by *Aspergillus niger*, *Botrytis cinerea*, *Penicillium expansum*, and *Rhizopus stolonifer* under both ambient and cold conditions. At lower concentrations (4 mg/mL chitosan with 1.25–2.5 μL/mL EO), the coatings maintained physicochemical and sensory quality without affecting consumer acceptance.

## 4 Conclusion

This study demonstrated the antibacterial mechanism of essential oil combinations and examined how emulsification parameters affect the particle size of nanoemulsions. Chemical composition analysis revealed that the major constituents were geraniol in Ci essential oil (48.04%), citral in Lc essential oil (20.36%), isobornyl acetate in Ca essential oil (21.17%), and D-limonene in Kl essential oil (64.15%). Among the EOs, Ca had the highest antioxidant activity, likely due to its high content of oxygenated compounds. On the other hand, CI was the most effective against *E. coli*. The combination of CiCa exhibited the strongest antibacterial effects, particularly against *E. coli*, and demonstrated enhanced radical scavenging activity in all antioxidant assays. Among the emulsification methods, the PI produced the smallest particle size (14.17 nm), the narrowest PDI (0.09), and the most stable zeta potential (−23 mV), resulting in highly uniform and stable nanoemulsions. These characteristics contributed to the superior antioxidant performance of the PI nanoemulsion. When incorporated into chitosan-based edible films, these nanoemulsions effectively delayed microbial growth, reduced pH and TVB-N fluctuations, limited lipid oxidation, and maintained the texture and color of MC during refrigerated storage. However, this study has certain limitations. The antimicrobial and antioxidant evaluations were conducted using a single meat model; the natural compositional variability of EO was not considered, and a sensory evaluation related to overall acceptability was not performed. However, a limitation of the present study is the absence of advanced analytical techniques to provide direct mechanistic evidence for the antimicrobial action. Methodologies such as electron microscopy (SEM, TEM) to visualize cellular damage, flow cytometry to assess membrane integrity, and biochemical assays to measure the leakage of intracellular enzymes like ATPase were not employed. The nanoemulsified CiCa prepared via the PI technique represents a potent and natural preservative system for meat products. The nanoemulsion-embedded chitosan film serves as an active packaging material, forming a protective barrier that prolongs shelf life. This approach not only enhances food safety but also aligns with current trends toward biodegradable, clean-label packaging solutions. Future studies are needed to evaluate the impact of EO composition variability on analysis reproducibility. Further research is also required regarding the long-term stability, migration properties, industrial-scale feasibility, and toxicology of the coatings across different food products, to ensure both the safety and consumer acceptability of nanoemulsion-embedded films.

## Supporting information

S1 File Providing a systematic evaluation of antioxidant capacities (ABTS, DPPH, Hydroxyl radical, and FRAP) and minimum inhibitory concentrations (MIC) for individual essential oils and their mixtures. Furthermore, it documents the practical application of nanoemulsions in maintaining the physicochemical stability, textural profile (TPA), and microbial safety of minced chicken samples over a 10-day storage period.
(XLSX)

S2 File Containing GC-MS chromatograms and detailed chemical constituent identifications for the essential oils of Citronella, Litsea cubeba, Coleus amboinicius, and Kaffir Lime. It also features a comprehensive series of kinetic growth curves illustrating the time-dependent inhibitory effects of various essential oil concentrations and mixtures against E. coli and L. plantarum.
(DOCX)

S1 Fig Graphical abstract. (TIFF)

## Abbreviations

ABTS: 2,2’-azino-bis(3-ethylbenzothiazoline-6-sulfonic acid) diammonium salt
C: control
Ca: *Coleus Amboinicus*
Ci: Citronella
DMSO: dimethyl sulfoxide
ECF: essential oil-loaded chitosan film
DPPH: 2,2-diphenyl-1-picrylhydrazyl
EDTA: ethylenediaminetetraacetic acid
EO: essential oil
EOM: essential oil mixture
FICI: fractional inhibitory concentration index
FRAP: ferric reducing antioxidant power
GC-MS: gas chromatography-mass spectrometry
HSH: high-speed shearing homogenization
Kl: Kaffir lime
LAB: lactic acid bacteria
Lc: *Litsea Cubeba*
MC: minced chicken
MDA: malonaldehyde
MIC: minimum inhibitory concentration
NE: non-emulsified essential oil
NHSHCF: nanoemulsion high-speed shearing homogenization chitosan film
NPICF: nanoemulsion phase inversion chitosan film
NUECF: nanoemulsion ultrasonic emulsification chitosan film
PDI: polydispersity index
PI: phase inversion
TBA: 2-thiobarbituric acid
TBARS: thiobarbituric acid reactive substances
TCA: trichloroacetic acid
TPA: texture profile analysis
TPTZ: 2,4,6-tris(2-pyridyl)-s-triazine
TVB-N: total volatile base nitrogen
TVC: total viable count
UE: ultrasonic emulsification
