# Supplementary material for: Applying nanoemulsions produced from the essential oil mixture of citronella and Coleus Amboinicus to preserve minced chicken meat
Source: PLoS One. 2026 Jan 5;21(1):e0339984. doi: 10.1371/journal.pone.0339984 (PMC12768258; doi:10.1371/journal.pone.0339984)
Supplement: S2 File — It also features a comprehensive series of kinetic growth curves illustrating the time-dependent inhibitory effects of various essential oil concentrations and mixtures against E. coli and L. plantarum. (DOCX) [file pone.0339984.s002.docx]

Applying nanoemulsions produced from the essential oil mixture of citronella and *Coleus Amboinicius* to preserve minced chicken meat

Tan Phat Vo^1,2*^, Nguyen Van Nhi Le^1,2^, Tan Triet Tcheng^1,2^, Le Song Tu Pham^1,2^, Tong Minh Quan Truong^1,2^, Ngoc Bao Vy Nguyen^1,2^, Dinh Quan Nguyen^1,2*^

^1^Laboratory of Biofuel and Biomass Research, Faculty of Chemical Engineering, Ho Chi Minh City University of Technology (HCMUT), 268 Ly Thuong Kiet Street, District 10, Ho Chi Minh City, Vietnam

^2^Vietnam National University Ho Chi Minh City, Linh Trung Ward, Thu Duc City, Ho Chi Minh City, Vietnam

^*^Corresponding author: [ndquan@hcmut.edu.vn](mailto:ndquan@hcmut.edu.vn) (Dinh Quan Nguyen), [phatbt9@gmail.com](mailto:phatbt9@gmail.com) (Tan Phat Vo)

A


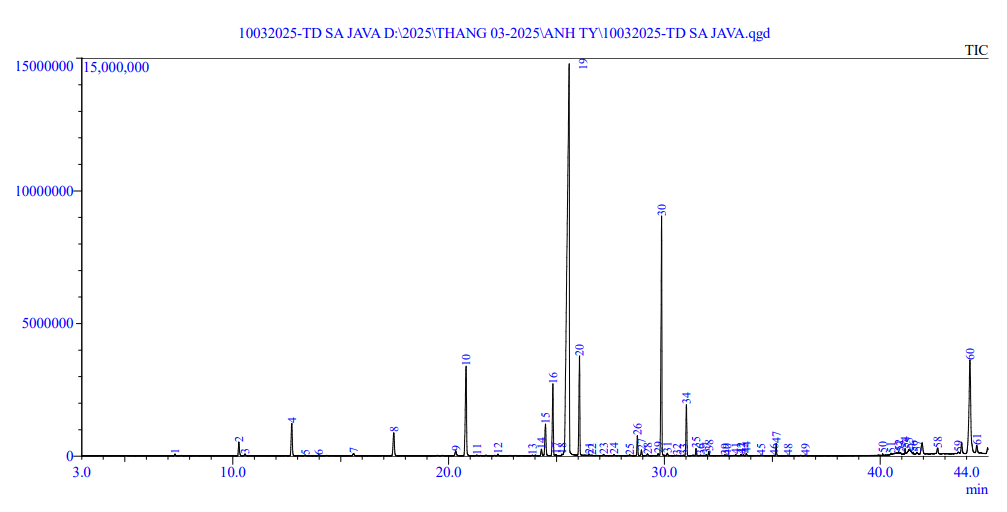


B

**
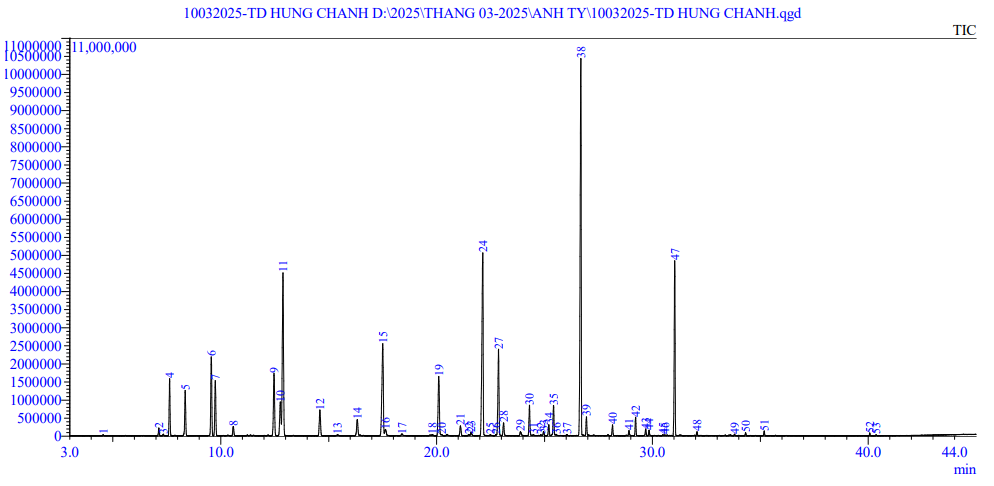
**

**Figure S1.** The GC-MS chromatograms of four essential oils; (A): The GC-MS chromatogram of citronella essential oil; (B): The GC-MS chromatogram of *Litsea Cubeba* essential oil; (C): The GC-MS chromatogram of *Coleus Amboinicus* essential oil; (D): The GC-MS chromatogram of kaffir lime essential oil.

C


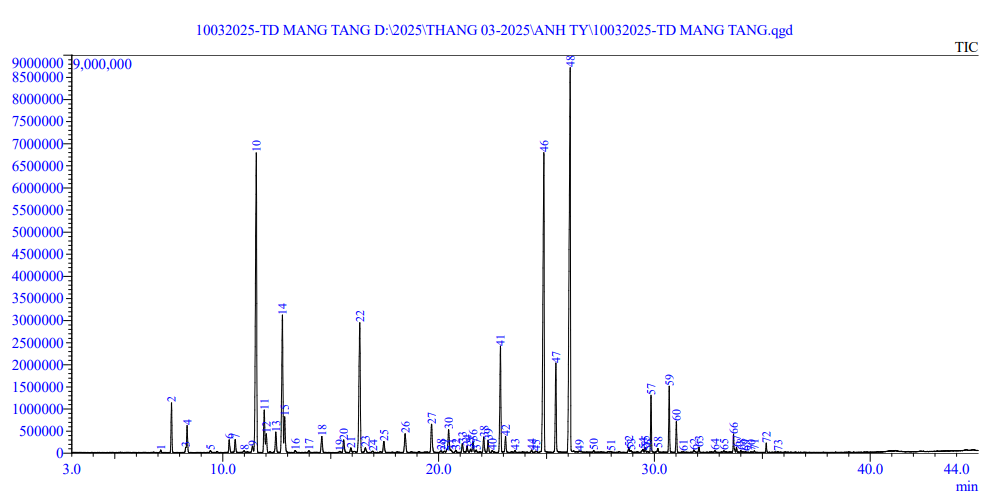


D


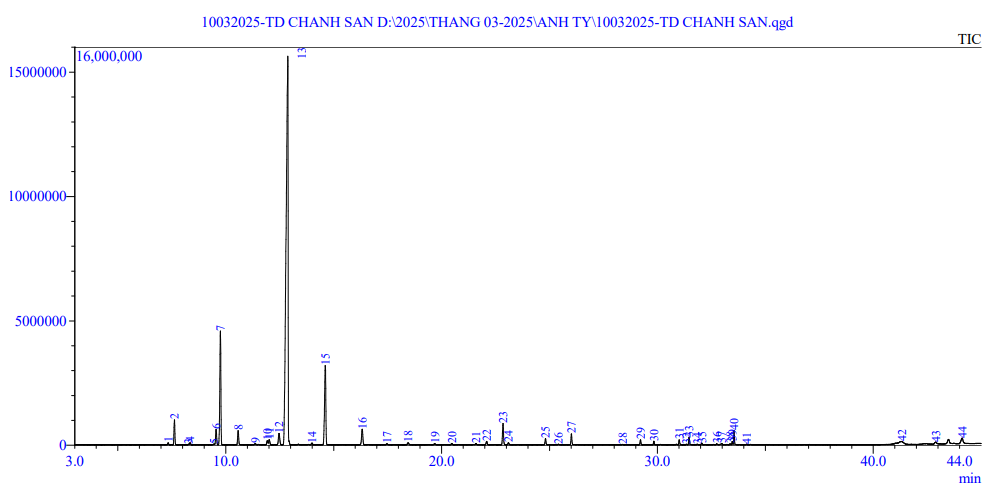


**Figure S1.** (continue)

**Table S1.** Chemical profile of Citronella essential oil

| **Peak** | **Retention Time** | **% Area** | **Name** | **Formula** | **M** | **Classification** |
| --- | --- | --- | --- | --- | --- | --- |
| 1 | 7.322 | 0.08 | .alpha.-Thujene | C_10_H_16_ | 136 | Monoterpenes |
| 2 | 10.280 | 0.76 | 6-Methyl-5-hepten-2-one | C_8_H_14_O | 126 | Ketones |
| 3 | 10.569 | 0.10 | .beta.-Myrcene | C_10_H_16_ | 136 | Monoterpenes |
| 4 | 12.733 | 2.03 | D-Limonene | C_10_H_16_ | 136 | Monoterpenes |
| 5 | 13.352 | 0.03 | trans-.beta.-Ocimene | C_10_H_16_ | 136 | Monoterpenes |
| 6 | 13.977 | 0.04 | 1,3,6-Octatriene, 3,7-dimethyl-, (Z)- | C_10_H_16_ | 136 | Monoterpenes |
| 7 | 15.598 | 0.14 | 4-Nonanone | C_9_H_18_O | 142 | Ketones |
| 8 | 17.459 | 1.64 | Linalool | C_10_H_18_O | 154 | Monoterpenes |
| 9 | 20.329 | 0.32 | ISOPULEGOL 2 | C_10_H_18_O | 154 | Monoterpenes |
| 10 | 20.808 | 6.02 | 6-Octenal, 3,7-dimethyl-, (R)- | C_10_H_18_O | 154 | Monoterpenoids |
| 11 | 21.317 | 0.02 | Isoneral | C_10_H_16_O | 152 | Monoterpenoids |
| 12 | 22.284 | 0.08 | Isogeranial | C_10_H_16_O | 152 | Monoterpenoids |
| 13 | 23.877 | 0.03 | (-)-cis-Isopiperitenol | C_10_H_16_O | 152 | Monoterpene derivatives |
| 14 | 24.300 | 0.37 | Nerol | C_10_H_18_O | 154 | Monoterpenes |
| 15 | 24.487 | 2.00 | .beta.-Citronellol | C_10_H_20_O | 156 | Monoterpenes |
| 16 | 24.833 | 3.83 | 2,6-Octadienal, 3,7-dimethyl-, (Z)- | C_10_H_16_O | 152 | Monoterpenes |
| 17 | 24.985 | 0.06 | 3,6-Octadien-1-ol, 3,7-dimethyl-, (Z)- | C_10_H_18_O | 154 | Aliphatic alcohols |
| 18 | 25.252 | 0.06 | 1,2,3-Propanetriol, 1-acetate | C_5_H_10_O_4_ | 134 | Glycerides |
| 19 | 25.583 | 48.04 | Geraniol | C_10_H_18_O | 154 | Monoterpenes |
| 20 | 26.064 | 4.97 | Citral | C_10_H_16_O | 152 | Monoterpenoids |
| 21 | 26.512 | 0.05 | Oxiranemethanol, 3-methyl-3-(4-methyl-3-pentenyl)- | C_10_H_18_O_2_ | 170 | Epoxides |
| 22 | 26.642 | 0.02 | Benzene, 1-methoxy-4-(1-propenyl)- (CAS) Anethole | C_10_H_12_O | 148 | Methoxybenzenes |
| 23 | 27.176 | 0.06 | 2,6-Octadien-1-ol, 3,7-dimethyl-, formate, (Z)- | C_11_H_18_O_2_ | 182 | Formates |
| 24 | 27.665 | 0.04 | 6-Octenoic acid, 3,7-dimethyl- | C_10_H_18_O_2_ | 170 | Monoterpenoids |
| 25 | 28.390 | 0.03 | iranemethanol, 3-methyl-3-(4-methyl-3-pentenyl)- (CAS) 2-ACETYL-3,3-DIMETHYL-2-(3-OXO-BUTYL)-CYCLOPENTANO | C_10_H_18_O_2_ | 170 | Epoxides |
| 26 | 28.745 | 0.87 | 1,2,3-Propanetriol, triacetate (CAS) Triacetin | C_9_H_14_O_6_ | 218 | Triglycerides |
| 27 | 28.934 | 0.27 | 6-Octen-1-ol, 3,7-dimethyl-, acetate | C_12_H_22_O_2_ | 198 | Monoterpenoids |
| 28 | 29.217 | 0.08 | NERYLACETATE | C_12_H_20_O_2_ | 196 | Monoterpenoids |
| 29 | 29.707 | 0.10 | Isopulegol acetate | C_12_H_20_O_2_ | 196 | Monoterpenoids |
| 30 | 29.868 | 11.83 | Geranyl acetate | C_12_H_20_O_2_ | 196 | Monoterpenoids |
| 31 | 30.138 | 0.12 | (-)-.beta.-Elemene | C_15_H_24_ | 204 | Sesquiterpenes |
| 32 | 30.568 | 0.01 | Bicyclo[7.2.0]undec-4-ene, 4,11,11-trimethyl-8-methylene-,[1R-(1R*,4Z,9S*)]- | C_15_H_24_ | 204 | Sesquiterpenes |
| 33 | 30.873 | 0.02 | trans-.alpha.-Bergamotene | C_15_H_24_ | 204 | Polycyclic olefins |
| 34 | 31.017 | 2.53 | TRANS(.BETA.)-CARYOPHYLLENE | C_15_H_24_ | 204 | Sesquiterpenes |
| 35 | 31.464 | 0.32 | .alpha.-Bergamotene | C_15_H_24_ | 204 | Polycyclic olefins |
| 36 | 31.670 | 0.02 | (1R,5R)-4-Methylene-1-((R)-6-methylhept-5-en-2-yl)bicyclo[3.1.0]hexane, (relative configuration) | C_15_H_24_ | 204 | Monoterpene derivatives |
| 37 | 31.937 | 0.03 | Naphthalene, decahydro-1,6-bis(methylene)-4-(1-methylethyl)-, (4.alpha.,4a.alpha.,8a.alpha.)- | C_15_H_24_ | 204 | Sesquiterpene derivatives |
| 38 | 32.057 | 0.20 | .alpha.-Humulene | C_15_H_24_ | 204 | Sesquiterpenes |
| 39 | 32.796 | 0.03 | .alpha.-Cubebene | C_15_H_24_ | 204 | Sesquiterpenes |
| 40 | 32.893 | 0.02 | (E)-.beta.-Famesene | C_15_H_24_ | 204 | Sesquiterpenes |
| 41 | 33.299 | 0.05 | .alpha.-Muurolene-(-) | C_15_H_24_ | 204 | Sesquiterpenes |
| 42 | 33.545 | 0.04 | .beta.-Bisabolene | C_15_H_24_ | 204 | Sesquiterpenes |
| 43 | 33.657 | 0.04 | Naphthalene, 1,2,3,4,4a,5,6,8a-octahydro-7-methyl-4-methylene-1-(1-methylethyl)-, (1.alpha.,4a.beta.,8a.alpha.)- | C_15_H_24_ | 204 | Sesquiterpenes |
| 44 | 33.796 | 0.09 | .delta.-Cadinene | C_15_H_24_ | 204 | Sesquiterpenes |
| 45 | 34.472 | 0.02 | 3,7-Cyclodecadiene-1-methanol, .alpha.,.alpha.,4,8-tetramethyl-, [s-(Z,Z)] | C_15_H_26_O | 222 | Sesquiterpenoids |
| 46 | 35.067 | 0.02 | (2E,4S,7E)-4-Isopropyl-1,7-dimethylcyclodeca-2,7-dienol | C_15_H_26_O | 222 | Alcohols |
| 47 | 35.177 | 0.48 | Caryophyllene oxide | C_15_H_24_O | 220 | Sesquiterpenes |
| 48 | 35.713 | 0.01 | (1R,3E,7E,11R)-1,5,5,8-Tetramethyl-12-oxabicyclo[9.1.0]dodeca-3,7-diene | C_15_H_24_O | 220 | Epoxides |
| 49 | 36.522 | 0.02 | .alpha.-Cadinol | C_15_H_26_O | 222 | Sesquiterpenoids |
| 50 | 40.110 | 0.04 | n-Hexadecanoic acid | C_16_H_32_O_2_ | 256 | Fatty acids |
| 51 | 40.496 | 0.05 | Naphthalene, 2-decyldecahydro- | C_20_H_38_ | 278 | Hydrocarbons |
| 52 | 40.853 | 0.03 | FARNESOL 1 | C_15_H_26_O | 222 | Sesquiterpenes |
| 53 | 40.926 | 0.05 | trans-Geranylgeraniol | C_20_H_34_O | 290 | Diterpenes |
| 54 | 41.149 | 0.14 | THIOGERANIOL | C_10_H_18_S | 170 | Monoterpenoids |
| 55 | 41.316 | 0.87 | .alpha.-Amyrin | C_30_H_50_O | 426 | Sesquiterpene derivatives |
| 56 | 41.525 | 0.07 | Cyclopropanemethanol, 2-methyl-2-(4-methyl-3-pentenyl)- | C_11_H_20_O | 168 | Alcohols |
| 57 | 41.694 | 0.17 | 2,6,10,14,18,22-Tetracosahexaene, 2,6,10,15,19,23-hexamethyl- (CAS) Squalene | C_30_H_50_ | 410 | Triterpenes |
| 58 | 42.659 | 0.36 | Widdrol hydroxyether | C_15_H_26_O_2_ | 238 | Alcohols |
| 59 | 43.616 | 0.11 | 17-Pentatriacontene | C_35_H_70_ | 490 | Hydrocarbons |
| 60 | 44.155 | 9.44 | Tetracontane | C_40_H_82_ | 562 | Hydrocarbons |
| 61 | 44.470 | 0.61 | Stigmasta-5,22-dien-3-ol, (3.beta.,22E)- (CAS) Stigmasterol | C_29_H_48_O | 412 | Sterols |

**Table S2.** Chemical profile of *Litsea Cubeba* essential oil

| **Peak** | **Retention Time** | **% Area** | **Name** | **Formula** | **M** | **Classification** |
| --- | --- | --- | --- | --- | --- | --- |
| 1 | 7.127 | 0.08 | Tricyclene | C_10_H_16_ | 136 | Monoterpenes |
| 2 | 7.624 | 1.76 | (1R)-2,6,6-Trimethylbicyclo[3.1.1]hept-2-ene | C_10_H_16_ | 136 | Monoterpenes |
| 3 | 8.280 | 0.16 | Bicyclo[2.2.1]heptane, 7,7-dimethyl-2-methylene- | C_10_H_16_ | 136 | Monoterpenes |
| 4 | 8.347 | 1.03 | Camphene | C_10_H_16_ | 136 | Monoterpenes |
| 5 | 9.427 | 0.07 | 1,3,5-Cycloheptatriene, 3,7,7-trimethyl- | C_10_H_14_ | 134 | Monoterpenoids |
| 6 | 10.298 | 0.53 | 6-Methyl-5-hepten-2-one | C_8_H_14_O | 126 | Ketones |
| 7 | 10.576 | 0.53 | .beta.-Myrcene | C_10_H_16_ | 136 | Monoterpenes |
| 8 | 10.994 | 0.08 | 2-Carene | C_10_H_16_ | 136 | Monoterpenes |
| 9 | 11.380 | 0.30 | 1-PHELLANDRENE | C_10_H_16_ | 136 | Monoterpenes |
| 10 | 11.552 | 15.17 | .DELTA.3-Carene | C_10_H_16_ | 136 | Monoterpenes |
| 11 | 11.919 | 1.93 | Isocineole | C_10_H_18_O | 154 | Monoterpenes |
| 12 | 12.015 | 0.81 | .ALPHA. TERPINENE | C_10_H_16_ | 136 | Monoterpenes |
| 13 | 12.457 | 0.94 | Benzene, 1-methyl-2-(1-methylethyl)- (CAS) 1-Methyl-2-isopropylbenzene | C_10_H_14_ | 134 | Terpenes |
| 14 | 12.763 | 6.82 | D-Limonene | C_10_H_16_ | 136 | Monoterpenes |
| 15 | 12.867 | 1.59 | EUCALYPTOL (1,8-CINEOLE) | C_10_H_18_O | 154 | Monoterpenes |
| 16 | 13.359 | 0.09 | .ALPHA.-PINENE, (-)- | C_10_H_16_ | 136 | Monoterpenes |
| 17 | 13.994 | 0.08 | 1,3,6-Octatriene, 3,7-dimethyl-, (Z)- | C_10_H_16_ | 136 | Monoterpenes |
| 18 | 14.589 | 0.78 | .gamma.-Terpinene | C_10_H_16_ | 136 | Monoterpenes |
| 19 | 15.417 | 0.05 | P-MENTH-1-ENE-3.8-DIOL (CIS?) | C_10_H_18_O_2_ | 170 | Monoterpenoids |
| 20 | 15.602 | 0.63 | 4-Nonanone | C_9_H_18_O | 142 | Ketones |
| 21 | 15.936 | 0.21 | Benzene, (2-methoxyethyl)- | C_9_H_12_O | 136 | Benzenes |
| 22 | 16.346 | 7.13 | (+)-4-Carene | C_10_H_16_ | 136 | Cycloalkene |
| 23 | 16.608 | 0.22 | Benzene, 2-ethenyl-1,3-dimethyl- (CAS) 2,6-Dimethylstyrene | C_10_H_12_ | 132 | Benzenes |
| 24 | 16.951 | 0.05 | 3-Methyl-2-(2-methyl-2-butenyl)-furan | C_10_H_14_O | 150 | Monoterpenoids |
| 25 | 17.463 | 0.57 | Linalool | C_10_H_18_O | 154 | Monoterpenoids |
| 26 | 18.449 | 1.01 | Fenchol | C_10_H_18_O | 154 | Monoterpenes |
| 27 | 19.674 | 1.45 | TERPINENE 1-OL | C_10_H_18_O | 154 | Monoterpenes |
| 28 | 20.114 | 0.07 | 3-Cyclohexene-1-carboxaldehyde | C_7_H_10_O | 110 | Aldehydes |
| 29 | 20.265 | 0.04 | 6-Octenal, 7-methyl-3-methylene- | C_10_H_16_O | 152 | Monoterpenoids |
| 30 | 20.464 | 1.28 | Cyclohexanol, 1-methyl-4-(1-methylethenyl)- | C_10_H_18_O | 154 | Monoterpenes |
| 31 | 20.600 | 0.13 | Bicyclo[2.2.1]heptan-2-ol, 2,3,3-trimethyl- | C_10_H_18_O | 154 | Monoterpenoids |
| 32 | 20.798 | 0.09 | 6-Octenal, 3,7-dimethyl-, (R)- | C_10_H_18_O | 154 | Monoterpenoids |
| 33 | 21.101 | 0.38 | Isoborneol | C_10_H_18_O | 154 | Monoterpenes |
| 34 | 21.319 | 0.27 | Isoneral | C_10_H_16_O | 152 | Monoterpenoids |
| 35 | 21.477 | 0.13 | Cyclohexanol, 1-methyl-4-(1-methylethenyl)- (CAS) .beta.-Terpineol | C_10_H_18_O | 154 | Monoterpenes |
| 36 | 21.590 | 0.48 | Bicyclo[2.2.1]heptan-2-ol, 1,7,7-trimethyl-, (1S-endo)- | C_10_H_18_O | 154 | Monoterpenes |
| 37 | 21.742 | 0.10 | 2-((3,3-Dimethyloxiran-2-yl)methyl)-3-methylfuran | C_10_H_14_O_2_ | 166 | Monoterpenoids |
| 38 | 22.089 | 0.69 | 3-Cyclohexen-1-ol, 4-methyl-1-(1-methylethyl)-, (R)- | C_10_H_18_O | 154 | Monoterpenoids |
| 39 | 22.293 | 0.52 | Isogeranial | C_10_H_16_O | 152 | Monoterpenoids |
| 40 | 22.488 | 0.08 | Benzenemethanol, 4-(1-methylethyl)- (CAS) P-CYMEN-.ALPHA.-OL | C_10_H_14_O | 150 | Monoterpenes |
| 41 | 22.865 | 4.56 | 3-Cyclohexene-1-methanol, .alpha.,.alpha.,4-trimethyl- (CAS) CYCLOHEXENE, 1-METHYL-4-(2-PROPANOL-2-YL)- | C_10_H_18_O | 154 | Monoterpenes |
| 42 | 23.099 | 0.61 | Cyclohexanol, 1-methyl-4-(1-methylethylidene)- | C_10_H_18_O | 154 | Monoterpenes |
| 43 | 23.539 | 0.06 | Decanal (CAS) n-Decanal | C_10_H_20_O | 156 | Aldehydes |
| 44 | 24.305 | 0.09 | Nerol | C_10_H_18_O | 154 | Monoterpenes |
| 45 | 24.501 | 0.03 | .beta.-Citronellol | C_10_H_20_O | 156 | Monoterpenes |
| 46 | 24.877 | 15.00 | 2,6-Octadienal, 3,7-dimethyl-, (Z)- | C_10_H_16_O | 152 | Monoterpenes |
| 47 | 25.432 | 3.54 | trans-Geraniol | C_10_H_18_O | 154 | Monoterpenes |
| 48 | 26.096 | 20.36 | Citral | C_10_H_16_O | 152 | Monoterpenes |
| 49 | 26.510 | 0.02 | Limonene oxide, trans- | C_10_H_16_O | 152 | Monoterpenoids |
| 50 | 27.184 | 0.06 | 2,6-Octadien-1-ol, 3,7-dimethyl-, formate, (Z)- | C_11_H_18_O_2_ | 182 | Carboxylic acids |
| 51 | 27.973 | 0.02 | trans-Geranic acid methyl ester | C_11_H_18_O_2_ | 182 | Monoterpenoids |
| 52 | 28.813 | 0.19 | Tricyclo[5.4.0.0(2,8)]undec-9-ene, 2,6,6,9-tetramethyl-, (1R,2S,7R,8R)- | C_15_H_24_ | 204 | Cyclic olefin |
| 53 | 28.904 | 0.05 | Geranic acid | C_10_H_16_O_2_ | 168 | Monoterpenoids |
| 54 | 29.467 | 0.14 | 1,2,4-Metheno-1H-indene, octahydro-1,7a-dimethyl-5-(1-methylethyl)-, [1S-(1.alpha.,2.alpha.,3a.beta.,4.alpha.,5.alpha.,7a.beta. | C_15_H_24_ | 204 | Sesquiterpenes derivatives |
| 55 | 29.574 | 0.15 | 1,2,4-Methenoazulene, decahydro-1,5,5,8a-tetramethyl-, [1S-(1.alpha.,2.alpha.,3a.beta.,4.alpha.,8a.beta.,9R*)]- (CAS) Longicycl | C_15_H_24_ | 204 | Cycloalkanes |
| 56 | 29.681 | 0.03 | .alfa.-Copaene | C_15_H_24_ | 204 | Cycloalkanes |
| 57 | 29.840 | 1.77 | Geranyl acetate | C_12_H_20_O_2_ | 196 | Monoterpenoids |
| 58 | 30.158 | 0.12 | 1,4-Methano-1H-indene, octahydro-4-methyl-8-methylene-7-(1-methylethyl)-, [1S-(1.alpha.,3a.beta.,4.alpha.,7.alpha.,7a.beta.)]- | C_15_H_24_ | 204 | Sesquiterpenoids |
| 59 | 30.683 | 2.31 | Longifolene | C_15_H_24_ | 204 | Sesquiterpenes |
| 60 | 31.016 | 1.10 | TRANS(.BETA.)-CARYOPHYLLENE | C_15_H_24_ | 204 | Sesquiterpenes |
| 61 | 31.343 | 0.01 | Germacrene B (CAS) 1,5-Cyclodecadiene, 1,5-dimethyl-8-(1-methylethylidene)-, (E,E)- | C_15_H_24_ | 204 | Sesquiterpenes |
| 62 | 31.820 | 0.08 | trans-Isoeugenol | C_10_H_12_O_2_ | 164 | Benzenes |
| 63 | 32.062 | 0.17 | Humulene | C_15_H_24_ | 204 | Sesquiterpenes |
| 64 | 32.802 | 0.05 | Germacrene D | C_15_H_24_ | 204 | Sesquiterpenes |
| 65 | 33.217 | 0.04 | (3S,3aR,3bR,4S,7R,7aR)-4-Isopropyl-3,7-dimethyloctahydro-1H-cyclopenta[1,3]cyclopropa[1,2]benzen-3-ol | C_15_H_26_O | 222 | Sesquiterpenes derivatives |
| 66 | 33.666 | 0.57 | Naphthalene, 1,2,3,4,4a,5,6,8a-octahydro-7-methyl-4-methylene-1-(1-methylethyl)-, (1.alpha.,4a.beta.,8a.alpha.)- | C_15_H_24_ | 204 | Sesquiterpenes |
| 67 | 33.802 | 0.14 | .delta.-Cadinene | C_15_H_24_ | 204 | Sesquiterpenes |
| 68 | 34.001 | 0.05 | (E)-1-Methyl-4-(6-methylhept-5-en-2-ylidene)cyclohex-1-ene | C_15_H_24_ | 204 | Sesquiterpenes |
| 69 | 34.223 | 0.01 | Naphthalene, 1,2,4a,5,6,8a-hexahydro-4,7-dimethyl-1-(1-methylethyl)-, [1S-(1.alpha.,4a.beta.,8a.alpha.)]- | C_15_H_24_ | 204 | Cyclic olefin |
| 70 | 34.468 | 0.02 | Cyclohexanemethanol, 4-ethenyl-.alpha.,.alpha.,4-trimethyl-3-(1-methylethenyl)-, [1R-(1.alpha.,3.alpha.,4.beta.)]- | C_15_H_26_O | 222 | Sesquiterpenes |
| 71 | 34.630 | 0.03 | Butanoic acid, 3,7-dimethyl-2,6-octadienyl ester, (E)- (CAS) Geranyl butyrate | C_14_H_24_O_2_ | 224 | Carboxylic esters |
| 72 | 35.180 | 0.27 | Caryophyllene oxide | C_15_H_24_O | 220 | Sesquiterpenes |
| 73 | 35.720 | 0.02 | (1R,3E,7E,11R)-1,5,5,8-Tetramethyl-12-oxabicyclo[9.1.0]dodeca-3,7-diene | C_15_H_24_O | 220 | Cyclic ethers |

**Table S3.** Chemical profile of *Coleus Amboinicus* essential oil

| **Peak** | **Retention Time** | **%**  **Area** | **Name** | **Formula** | **M** | **Classification** |
| --- | --- | --- | --- | --- | --- | --- |
| 1 | 4.550 | 0.04 | Butanoic acid, 2-methyl-, ethyl ester (CAS) Ethyl 2-methylbutyrate | C_7_H_4_O_2_ | 130 | Fatty esters |
| 2 | 7.129 | 0.35 | Tricyclene | C_10_H_16_ | 136 | Monoterpenes |
| 3 | 7.338 | 0.07 | .alpha.-Thujene | C_10_H_16_ | 136 | Monoterpenes |
| 4 | 7.629 | 2.65 | (1R)-2,6,6-Trimethylbicyclo[3.1.1]hept-2-ene | C_10_H_16_ | 136 | Monoterpenes |
| 5 | 8.351 | 2.34 | Camphene | C_10_H_16_ | 136 | Monoterpenes |
| 6 | 9.560 | 4.19 | Sabinene | C_10_H_16_ | 136 | Monoterpenes |
| 7 | 9.746 | 2.98 | Bicyclo[3.1.1]heptane, 6,6-dimethyl-2-methylene-, (1S)- | C_10_H_16_ | 136 | Monoterpenes |
| 8 | 10.579 | 0.52 | .beta.-Myrcene | C_10_H_16_ | 136 | Monoterpenes |
| 9 | 12.467 | 3.89 | Benzene, 1-methyl-2-(1-methylethyl)- (CAS) 1-Methyl-2-isopropylbenzene | C_10_H_14_ | 134 | Monoterpenes |
| 10 | 12.758 | 2.24 | D-Limonene | C_10_H_16_ | 136 | Monoterpenes |
| 11 | 12.882 | 10.36 | EUCALYPTOL (1,8-CINEOLE) | C_10_H_18_O | 154 | Monoterpenes |
| 12 | 14.592 | 1.69 | .gamma.-Terpinene | C_10_H_16_ | 136 | Monoterpenes |
| 13 | 15.403 | 0.04 | 2-Furanmethanol, 5-ethenyltetrahydro-.alpha.,.alpha.,5-trimethyl-, cis- | C_10_H_18_O_2_ | 170 | Cyclohexanols |
| 14 | 16.324 | 1.07 | .ALPHA.-TERPINOLENE | C_10_H_16_ | 136 | Monoterpenes |
| 15 | 17.504 | 6.98 | Linalool | C_10_H_18_O | 154 | Monoterpenes |
| 16 | 17.627 | 0.49 | Bicyclo[3.1.0]hexan-3-one, 4-methyl-1-(1-methylethyl)-, [1S-(1.alpha.,4.beta.,5.alpha.)]- | C_10_H_16_O | 152 | Monoterpene derivatives |
| 17 | 18.395 | 0.10 | Thujone | C_10_H_16_O | 152 | Monoterpenes |
| 18 | 19.808 | 0.06 | 1,2-Dihydrolinalool | C_10_H_20_O | 156 | Fatty alcohols |
| 19 | 20.099 | 3.99 | (+)-2-Bornanone | C_10_H_16_O | 152 | Monoterpenoids |
| 20 | 20.230 | 0.07 | Plinol D, (+)- | C_10_H_18_O | 154 | Alcohols |
| 21 | 21.103 | 0.60 | Isoborneol | C_10_H_18_O | 154 | Monoterpenes |
| 22 | 21.490 | 0.07 | Cyclohexanol, 1-methyl-4-(1-methylethenyl)- (CAS) .beta.-Terpineol | C_10_H_18_O | 154 | Monoterpenes |
| 23 | 21.598 | 0.21 | Bicyclo[2.2.1]heptan-2-ol, 1,7,7-trimethyl-, (1S-endo)- | C_10_H_18_O | 154 | Monoterpenes |
| 24 | 22.138 | 12.04 | 3-Cyclohexen-1-ol, 4-methyl-1-(1-methylethyl)-, (R)- | C_10_H_18_O | 154 | Monoterpenoids |
| 25 | 22.486 | 0.03 | Benzenemethanol, 4-(1-methylethyl)- (CAS) P-CYMEN-.ALPHA.-OL | C_10_H_14_O | 150 | Alcohols |
| 26 | 22.747 | 0.05 | ENDO-ISOCAMPHONONE | C_10_H_16_O | 152 | Alcohols |
| 27 | 22.869 | 4.90 | 3-Cyclohexene-1-methanol, .alpha.,.alpha.,4-trimethyl- (CAS) CYCLOHEXENE, 1-METHYL-4-(2-PROPANOL-2-YL)- | C_10_H_18_O | 154 | Monoterpenoids |
| 28 | 23.102 | 0.70 | Cyclohexanol, 1-methyl-4-(1-methylethylidene)- | C_10_H_18_O | 154 | Monoterpenoids |
| 29 | 23.884 | 0.26 | Ethanol, 2-phenoxy- (CAS) 2-Phenoxyethanol | C_8_H_10_O_2_ | 138 | Glycols |
| 30 | 24.301 | 1.54 | Nerol | C_10_H_18_O | 154 | Monoterpenes |
| 31 | 24.481 | 0.05 | Bicyclo[2.2.1]heptan-2-ol, 1,3,3-trimethyl-, acetate, (1S-exo)- | C_12_H_20_O_2_ | 196 | Monoterpenoids |
| 32 | 24.827 | 0.03 | 2,6-Octadienal, 3,7-dimethyl-, (Z)- | C_10_H_16_O | 152 | Monoterpenes |
| 33 | 24.956 | 0.20 | 2-Cyclohexen-1-one, 2-methyl-5-(1-methylethenyl)-, (S)- (CAS) d-Carvone | C_10_H_14_O | 150 | Monoterpenoids |
| 34 | 25.197 | 0.54 | p-Menth-8-en-3-ol, acetate | C_12_H_20_O_2_ | 196 | Monoterpenoids |
| 35 | 25.418 | 1.57 | trans-Geraniol | C_10_H_18_O | 154 | Monoterpenes |
| 36 | 25.540 | 0.05 | 2-(1-Cyclopent-1-enyl-1-methylethyl)cyclopentanone | C_13_H_20_O | 192 | Ketones |
| 37 | 26.037 | 0.05 | Citral | C_10_H_16_O | 152 | Monoterpenoids |
| 38 | 26.682 | 21.17 | Isobornyl acetate | C_12_H_20_O_2_ | 196 | Monoterpenes |
| 39 | 26.940 | 0.83 | Phenol, 5-methyl-2-(1-methylethyl)- (CAS) Thymol | C_10_H_14_O | 150 | Monoterpenes |
| 40 | 28.149 | 0.47 | Acetic acid, 1,7,7-trimethyl-bicyclo[2.2.1]hept-2-yl ester | C_12_H_20_O_2_ | 196 | Monoterpenes |
| 41 | 28.912 | 0.23 | Eugenol | [C_10_H_12_O](https://pubchem.ncbi.nlm.nih.gov/#query=C12H14O3)_2_ | 164 | Monoterpenes |
| 42 | 29.219 | 0.80 | NERYLACETATE | C_12_H_20_O_2_ | 196 | Monoterpenoids |
| 43 | 29.685 | 0.31 | .alpha.-Copaene | C_15_H_24_ | 204 | Sesquiterpenes |
| 44 | 29.842 | 0.23 | Geranyl acetate | C_12_H_20_O_2_ | 196 | Monoterpenoids |
| 45 | 30.488 | 0.04 | Benzene, 1,2-dimethoxy-4-(2-propenyl)- (CAS) Methyleugenol | C_11_H_14_O_2_ | 178 | Caffeic acids |
| 46 | 30.574 | 0.02 | CIS-CARYOPHYLLENE | C_15_H_24_ | 204 | Sesquiterpenes |
| 47 | 31.032 | 8.25 | TRANS(.BETA.)-CARYOPHYLLENE | C_15_H_24_ | 204 | Sesquiterpenes |
| 48 | 32.063 | 0.18 | Humulene | C_15_H_24_ | 204 | Sesquiterpenes |
| 49 | 33.804 | 0.06 | .delta.-Cadinene | C_15_H_24_ | 204 | Sesquiterpenes |
| 50 | 34.316 | 0.11 | Cyclohexene, 4-[(1E)-1,5-dimethyl-1,4-hexadien-1-yl]-1-methyl- | C_15_H_24_ | 204 | Sesquiterpenes |
| 51 | 35.179 | 0.19 | Caryophyllene oxide | C_15_H_24_O | 220 | Sesquiterpenes |
| 52 | 40.067 | 0.07 | m-Camphorene | C_20_H_32_ | 272 | Cycloalkene |
| 53 | 40.361 | 0.02 | p-Camphorene | C_20_H_32_ | 272 | Cycloalkene |

**Table S4.** Chemical profile of Kaffir Lime essential oil

| **Peak** | **Retention Time** | **% Area** | **Name** | **Formula** | **M** | **Classification** |
| --- | --- | --- | --- | --- | --- | --- |
| 1 | 7.332 | 0.14 | .alpha.-Thujene | C_10_H_16_ | 136 | Monoterpenes |
| 2 | 7.621 | 1.83 | (1R)-2,6,6-Trimethylbicyclo[3.1.1]hept-2-ene | C_10_H_16_ | 136 | Monoterpenes |
| 3 | 8.270 | 0.05 | Bicyclo[2.2.1]heptane, 7,7-dimethyl-2-methylene- | C_10_H_16_ | 136 | Monoterpenes |
| 4 | 8.346 | 0.16 | Camphene | C_10_H_16_ | 136 | Monoterpenes |
| 5 | 9.456 | 0.05 | 2H-Pyran, 2-ethenyltetrahydro-2,6,6-trimethyl- | C_10_H_18_O | 154 | Oxanes |
| 6 | 9.551 | 1.31 | Sabinene | C_10_H_16_ | 136 | Monoterpenes |
| 7 | 9.752 | 9.82 | Bicyclo[3.1.1]heptane, 6,6-dimethyl-2-methylene-, (1S)- | C_10_H_16_ | 136 | Monoterpenes |
| 8 | 10.573 | 1.19 | .beta.-Myrcene | C_10_H_16_ | 136 | Monoterpenes |
| 9 | 11.364 | 0.11 | 1-PHELLANDRENE | C_10_H_16_ | 136 | Monoterpenes |
| 10 | 11.922 | 0.38 | 7-Oxabicyclo[2.2.1]heptane, 1-methyl-4-(1-methylethyl)- | C_10_H_18_O | 154 | Monoterpenes |
| 11 | 12.013 | 0.50 | .ALPHA. TERPINENE | C_10_H_16_ | 136 | Monoterpenes |
| 12 | 12.469 | 1.31 | Benzene, 1-methyl-2-(1-methylethyl)- (CAS) 1-Methyl-2-isopropylbenzene | C_10_H_14_ | 134 | Benzenes |
| 13 | 12.874 | 64.15 | D-Limonene | C_10_H_16_ | 136 | Monoterpenes |
| 14 | 13.992 | 0.17 | 1,3,6-Octatriene, 3,7-dimethyl-, (E)- (CAS) .BETA. OCIMENE Y | C_10_H_16_ | 136 | Monoterpenes |
| 15 | 14.610 | 8.31 | .gamma.-Terpinene | C_10_H_16_ | 136 | Monoterpenes |
| 16 | 16.319 | 1.65 | (+)-4-Carene | C_10_H_16_ | 136 | Cyclic olefin |
| 17 | 17.467 | 0.10 | Linalool | C_10_H_18_O | 154 | Monoterpenes |
| 18 | 18.446 | 0.22 | Fenchol | C_10_H_18_O | 154 | Monoterpenes |
| 19 | 19.684 | 0.11 | TERPINENE 1-OL | C_10_H_18_O | 154 | Monoterpenes |
| 20 | 20.470 | 0.12 | Cyclohexanol, 1-methyl-4-(1-methylethenyl)- (CAS) .beta.-Terpineol | C_10_H_18_O | 154 | Monoterpenes |
| 21 | 21.592 | 0.12 | Bicyclo[2.2.1]heptan-2-ol, 1,7,7-trimethyl-, (1S-endo)- | C_10_H_18_O | 154 | Monoterpenes |
| 22 | 22.086 | 0.29 | 3-Cyclohexen-1-ol, 4-methyl-1-(1-methylethyl)-, (R)- | C_10_H_18_O | 154 | Monoterpenoids |
| 23 | 22.844 | 1.79 | 3-Cyclohexene-1-methanol, .alpha.,.alpha.,4-trimethyl- (CAS) CYCLOHEXENE, 1-METHYL-4-(2-PROPANOL-2-YL)- | C_10_H_18_O | 154 | Monoterpenes |
| 24 | 23.098 | 0.16 | Cyclohexanol, 1-methyl-4-(1-methylethylidene)- | C_10_H_18_O | 154 | Monoterpenes |
| 25 | 24.812 | 0.47 | 2,6-Octadienal, 3,7-dimethyl-, (Z)- | C_10_H_16_O | 152 | Monoterpenes |
| 26 | 25.405 | 0.03 | Geraniol | C_10_H_18_O | 154 | Monoterpenes |
| 27 | 26.013 | 0.82 | Citral | C_10_H_16_O | 152 | Monoterpenes |
| 28 | 28.382 | 0.03 | Cyclohexene, 4-ethenyl-4-methyl-3-(1-methylethenyl)-1-(1-methylethyl)-, (3R-trans)- | C_15_H_24_ | 204 | Sesquiterpenoids |
| 29 | 29.214 | 0.33 | NERYLACETATE | C_12_H_20_O_2_ | 196 | Monoterpenoids |
| 30 | 29.835 | 0.24 | Geranyl acetate | C_12_H_20_O_2_ | 196 | Monoterpenoids |
| 31 | 31.008 | 0.37 | CIS-CARYOPHYLLENE | C_15_H_24_ | 204 | Sesquiterpenoids |
| 32 | 31.340 | 0.03 | Germacrene B (CAS) 1,5-Cyclodecadiene, 1,5-dimethyl-8-(1-methylethylidene)-, (E,E)- | C_15_H_24_ | 204 | Sesquiterpenes |
| 33 | 31.464 | 0.48 | trans-.alpha.-Bergamotene | C_15_H_24_ | 204 | Cyclic olefin |
| 34 | 31.840 | 0.02 | Z-Citral | C_10_H_16_O | 152 | Monoterpenes |
| 35 | 32.054 | 0.12 | Farnesol | C_15_H_26_O | 222 | Alcohols |
| 36 | 32.748 | 0.06 | Geranyl vinyl ether | C_12_H_20_O | 180 | Ether |
| 37 | 32.976 | 0.08 | .delta.-Selinene | C_15_H_24_ | 204 | Sesquiterpenes |
| 38 | 33.366 | 0.06 | Cyclohexene, 4-[(1E)-1,5-dimethyl-1,4-hexadien-1-yl]-1-methyl- | C_15_H_24_ | 204 | Sesquiterpenes |
| 39 | 33.463 | 0.20 | .alpha.-Farnesene | C_15_H_24_ | 204 | Sesquiterpenes |
| 40 | 33.549 | 0.82 | .beta.-Bisabolene | C_15_H_24_ | 204 | Sesquiterpenes |
| 41 | 34.134 | 0.04 | 1H-Cyclopropa[a]naphthalene, 1a,2,3,3a,4,5,6,7b-octahydro-1,1,3a,7-tetramethyl-, [1aR-(1a.alpha.,3a.alpha.,7b.alpha.)]- | C_15_H_24_ | 204 | Sesquiterpenoids |
| 42 | 41.329 | 0.53 | .alpha.-Amyrin | C_30_H_50_O | 426 | Sesquiterpenes derivatives |
| 43 | 42.899 | 0.31 | 9,19-Cycloergost-24(28)-en-3-ol, 4,14-dimethyl-, acetate, (3.beta.,4.alpha.,5.alpha.)- | C_32_H_52_O_2_ | 468 | Triterpenoids |
| 44 | 44.117 | 0.89 | Tetracontane | C_40_H_82_ | 562 | Alkanes |


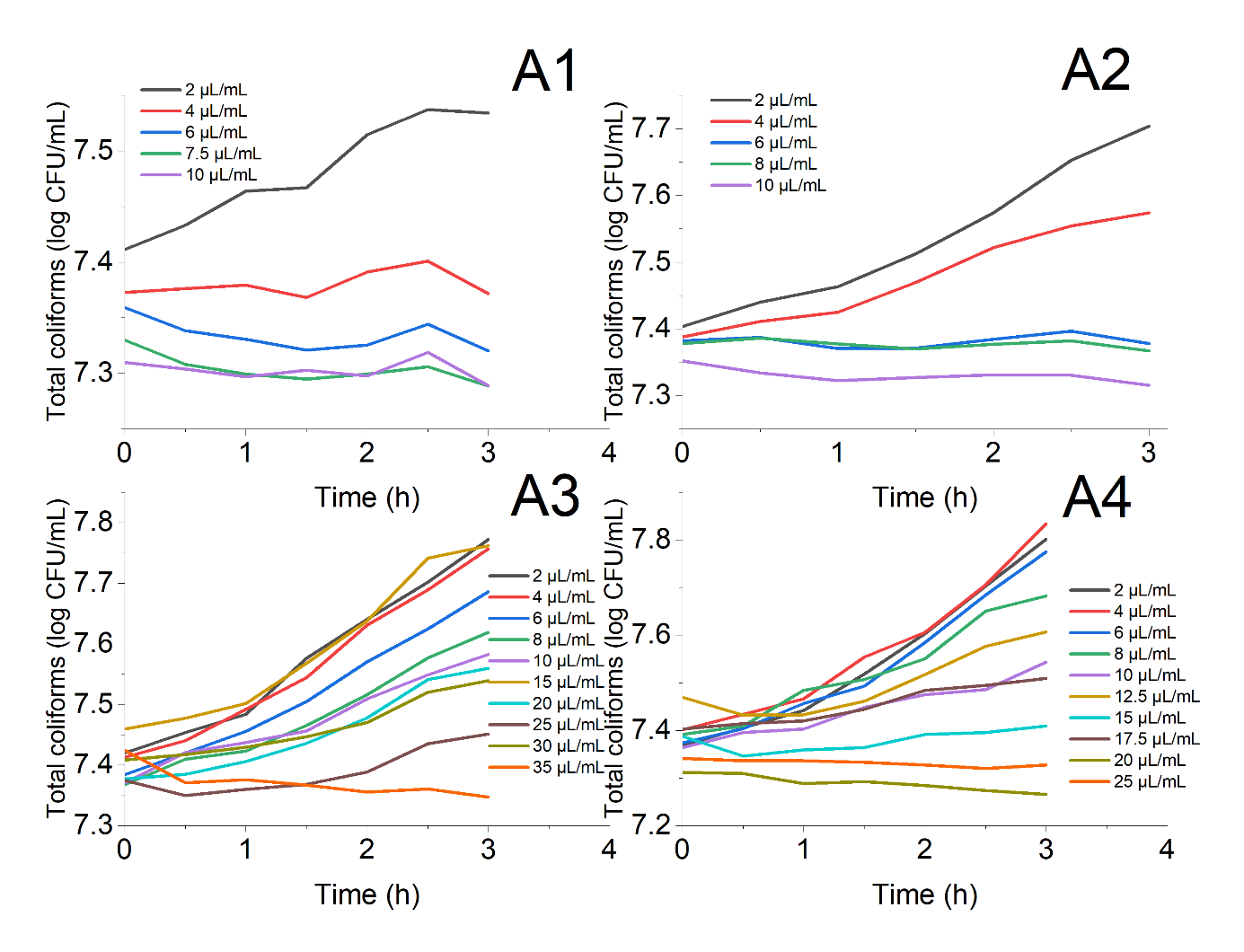


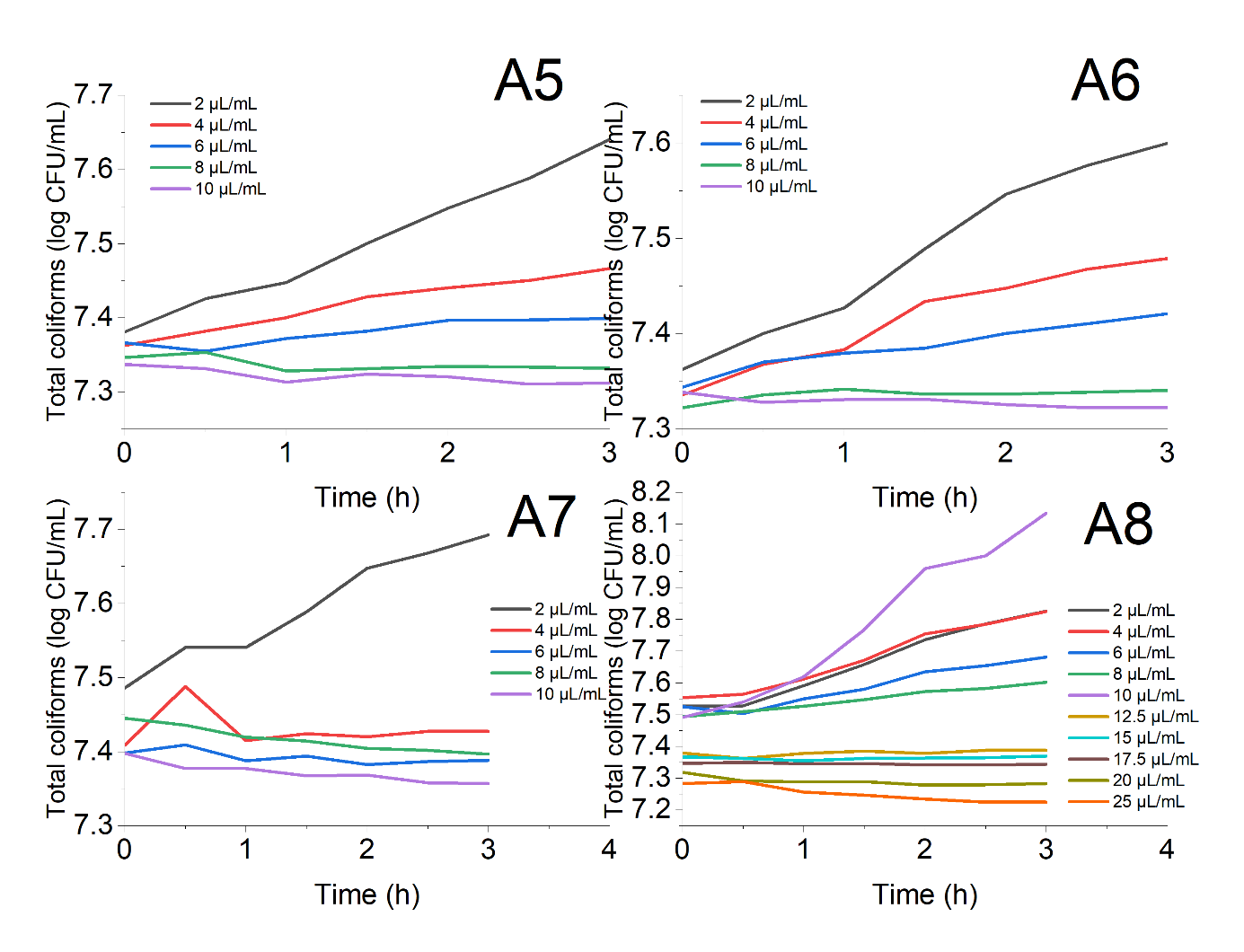


**Figure S2.** The minimum inhibitory concentration results (μL/mL) of four essential oils and their mixtures against *E.Coli*. (A1): MIC result of Ci; (A2): MIC result of Lc; (A3): MIC result of Ca; (A4): MIC result of Kl; (A5): MIC result of CiLc; (A6): MIC result of CiCa; (A7): MIC result of CiKl; (A8): MIC result of LcCa; (A9): MIC result of LcKl; (A10): MIC result of CaKl; (A11): MIC result of CiLcCa; (A12): MIC result of CiLcKl; (A13): MIC result of CiCaKl; (A14): MIC result of LcCaKl; (A15): MIC result of CiLcCaKl; (A16): MIC result of Penicillin.


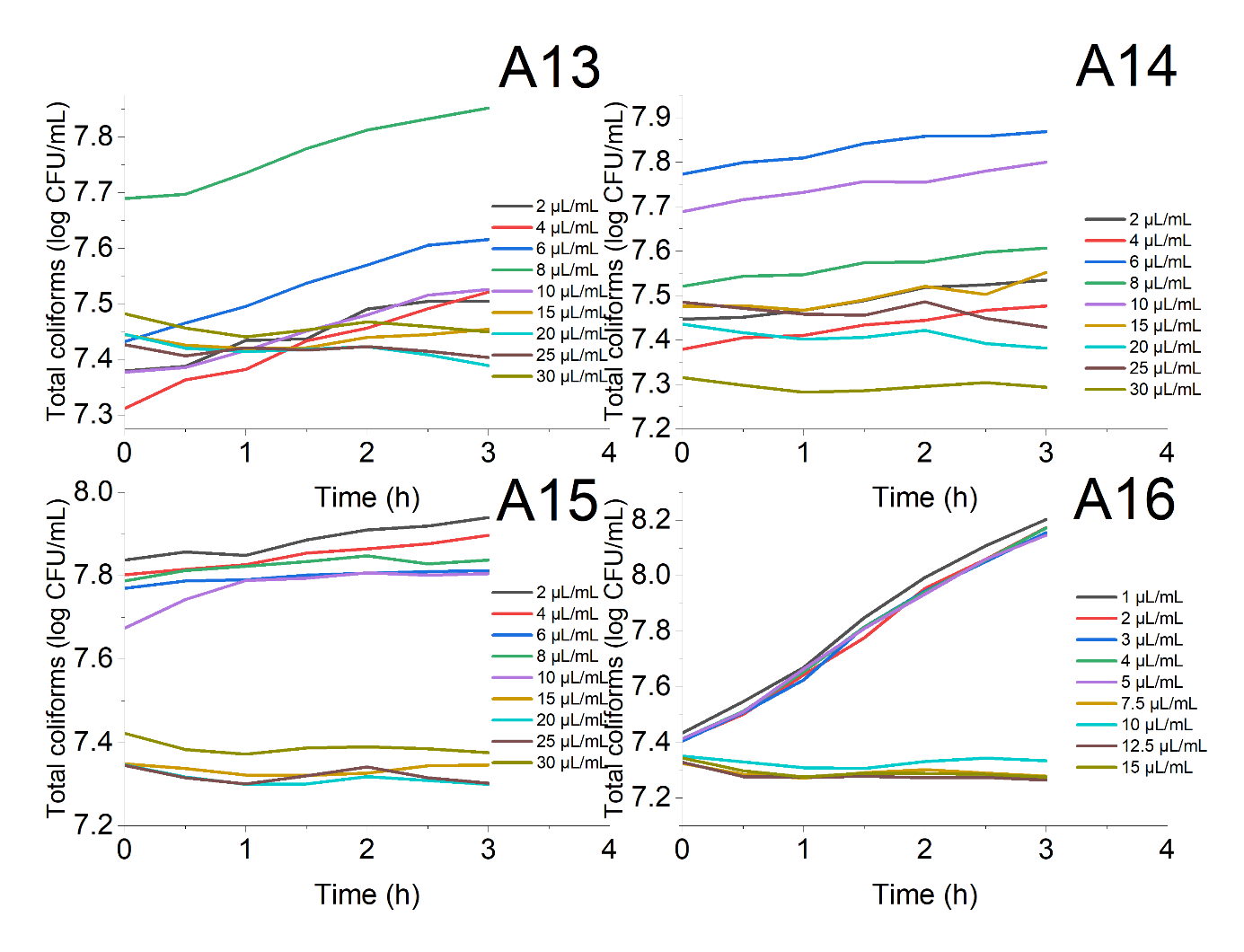

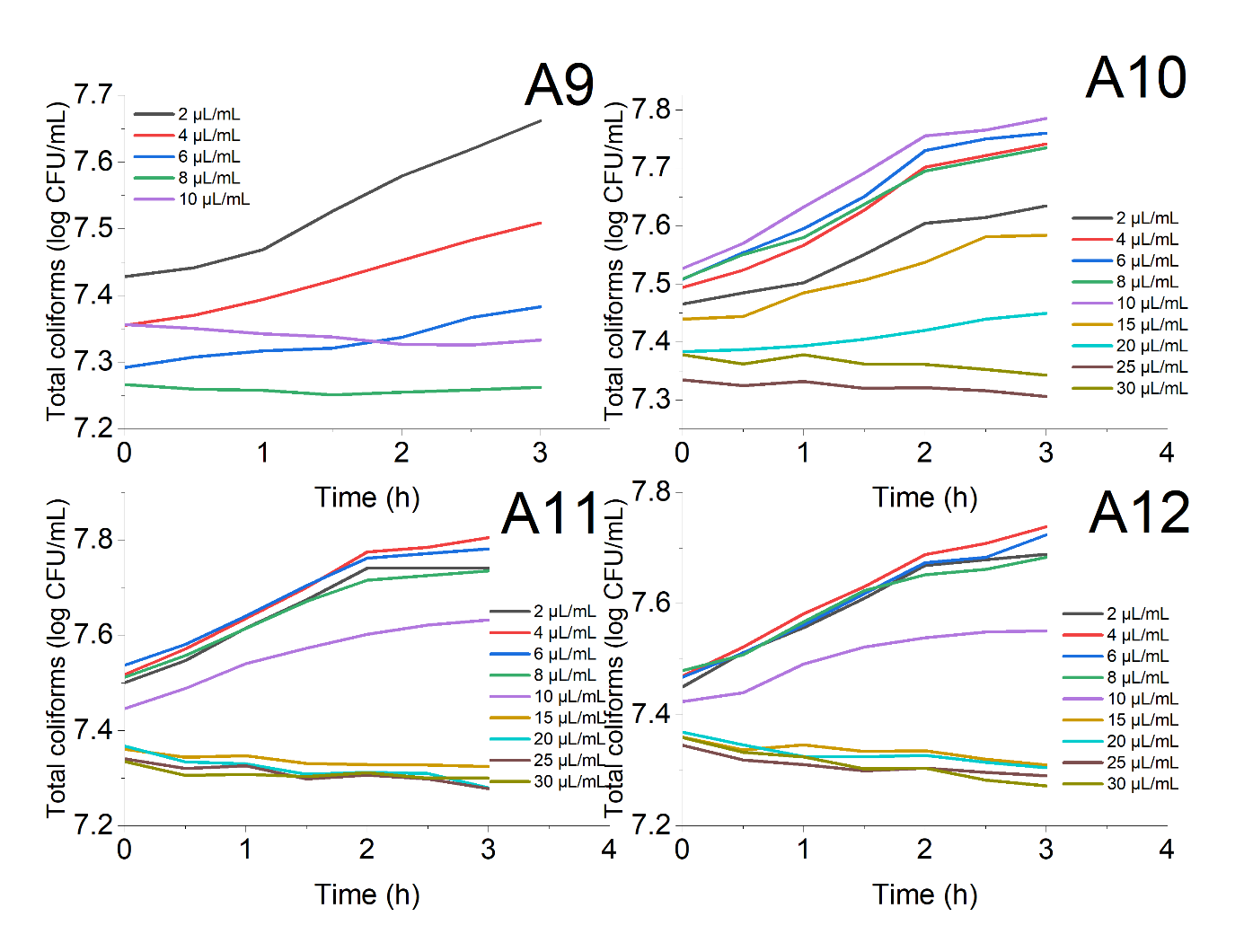


**Figure S2** (continue)


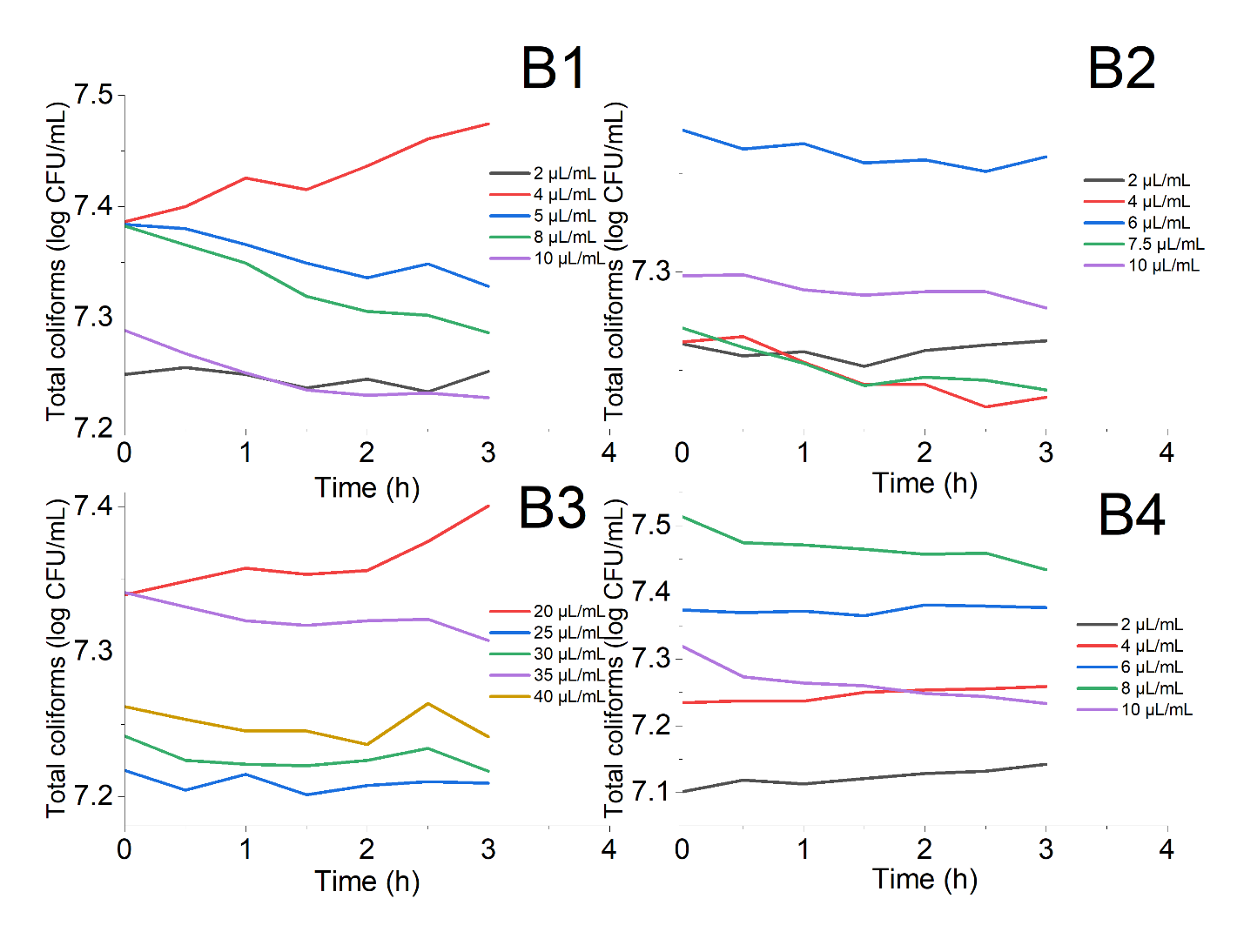

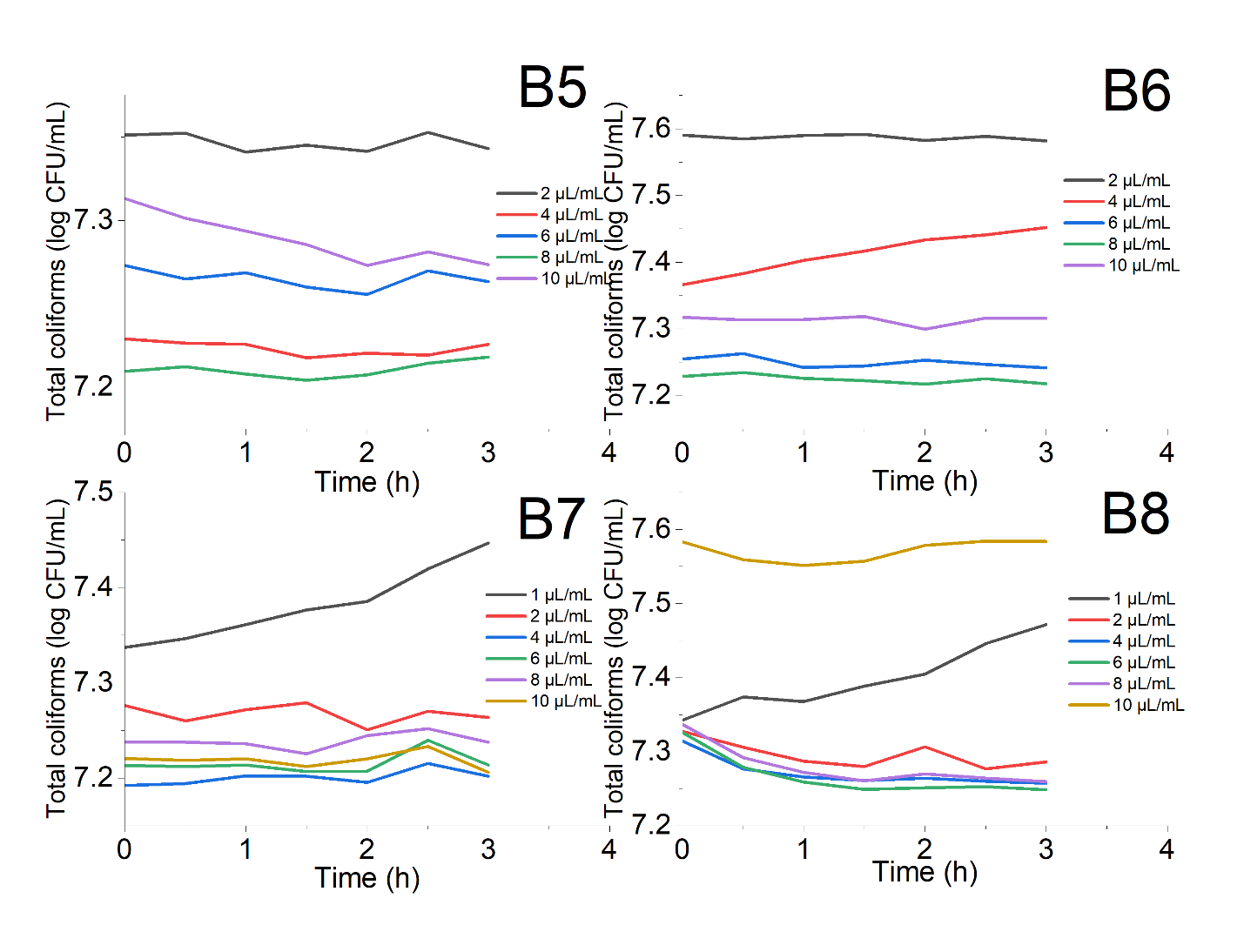


**Figure S3.** The minimum inhibitory concentration results (μL/mL) of four essential oils and their mixtures against *L.plantarum*. (B1): MIC result of Ci; (B2): MIC result of Lc; (B3): MIC result of Ca; (B4): MIC result of Kl; (B5): MIC result of CiLc; (B6): MIC result of CiCa; (B7): MIC result of CiKl; (B8): MIC result of LcCa; (B9): MIC result of LcKl; (B10): MIC result of CaKl; (B11): MIC result of CiLcCa; (B12): MIC result of CiLcKl; (B13): MIC result of CiCaKl; (B14): MIC result of LcCaKl; (B15): MIC result of CiLcCaKl; (B16): MIC result of Penicillin.


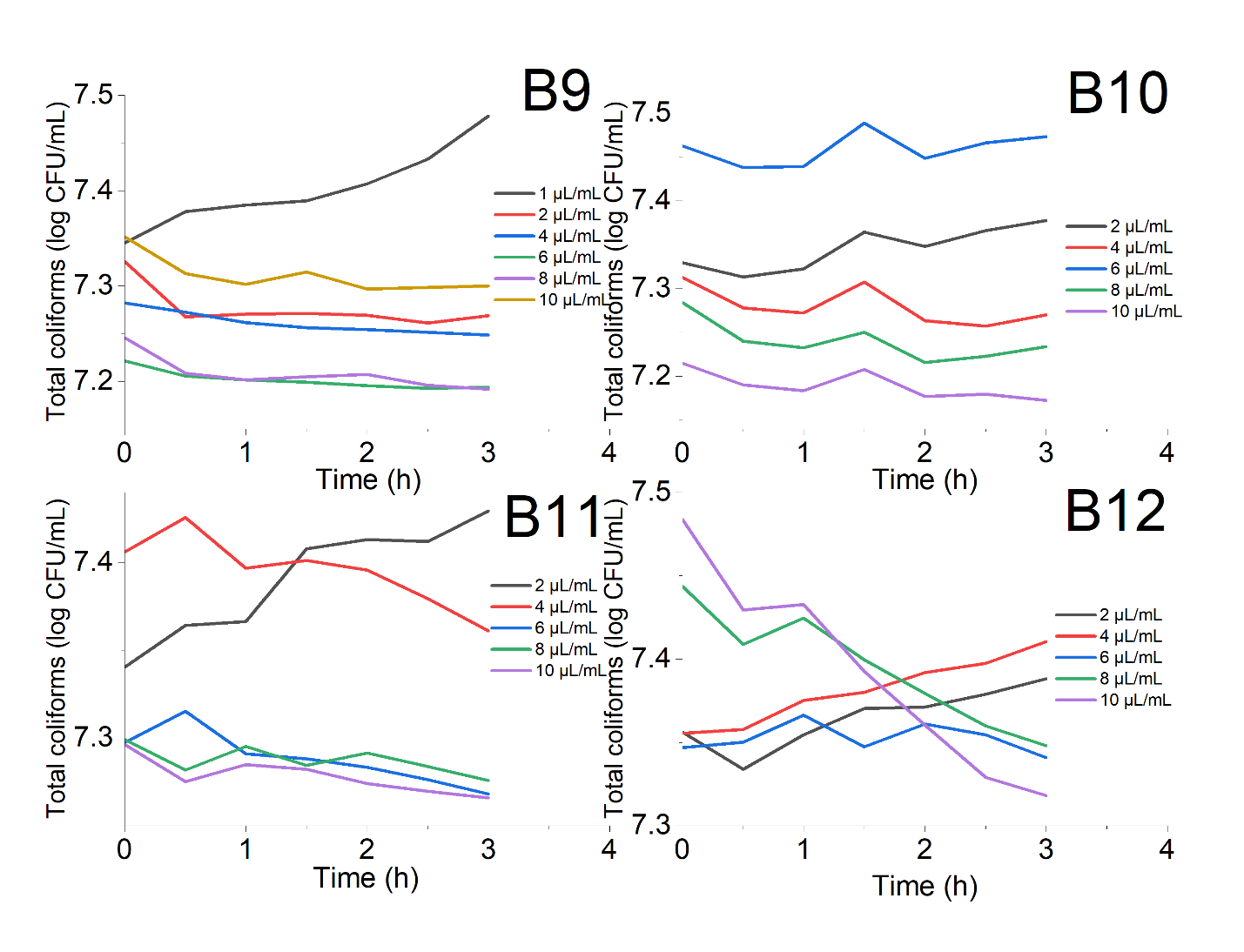


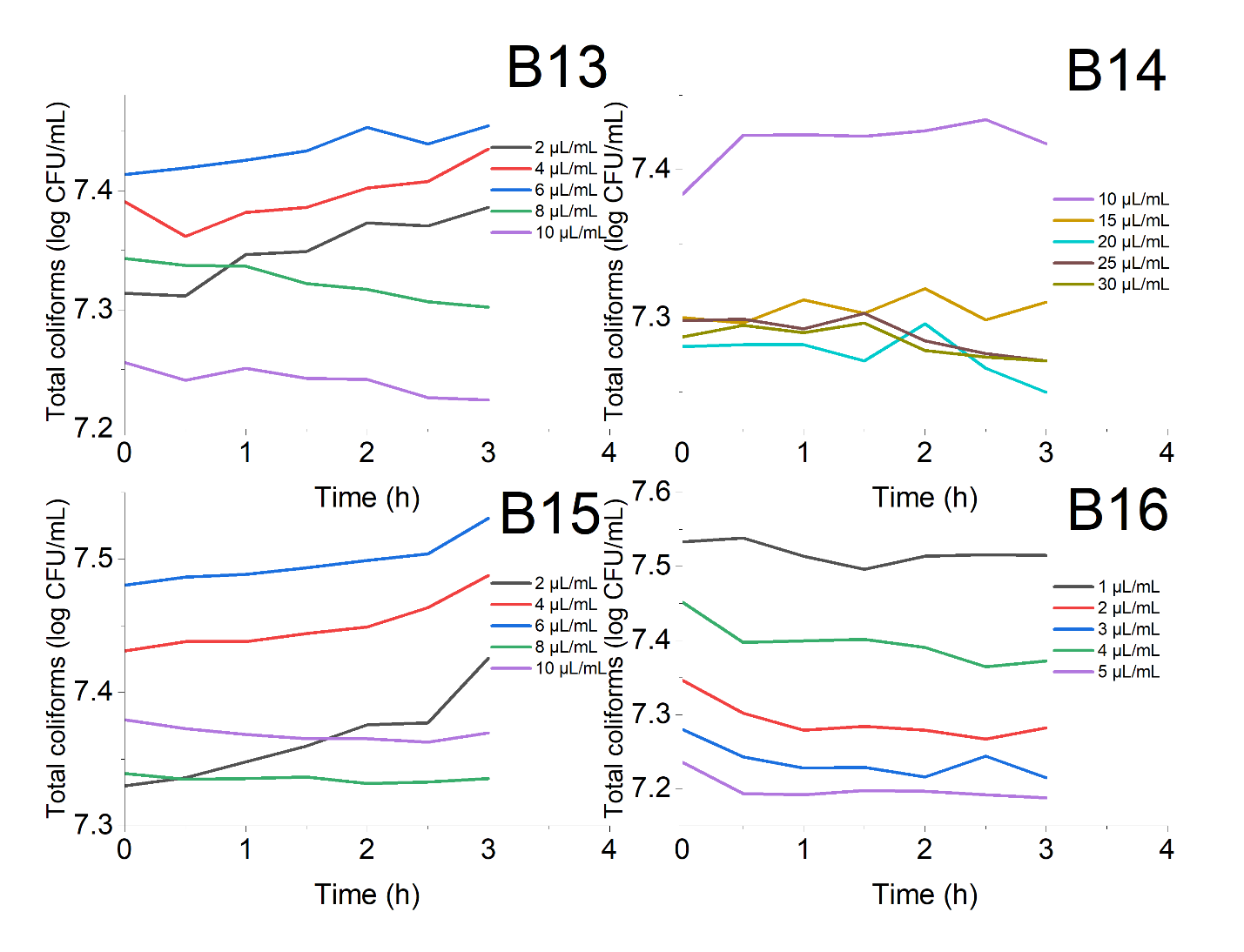


**Figure S3** (continue)
