# Supplementary figures and images for: Applying nanoemulsions produced from the essential oil mixture of citronella and Coleus Amboinicus to preserve minced chicken meat
Source: PLoS One. 2026 Jan 5;21(1):e0339984. doi: 10.1371/journal.pone.0339984 (PMC12768258; doi:10.1371/journal.pone.0339984)

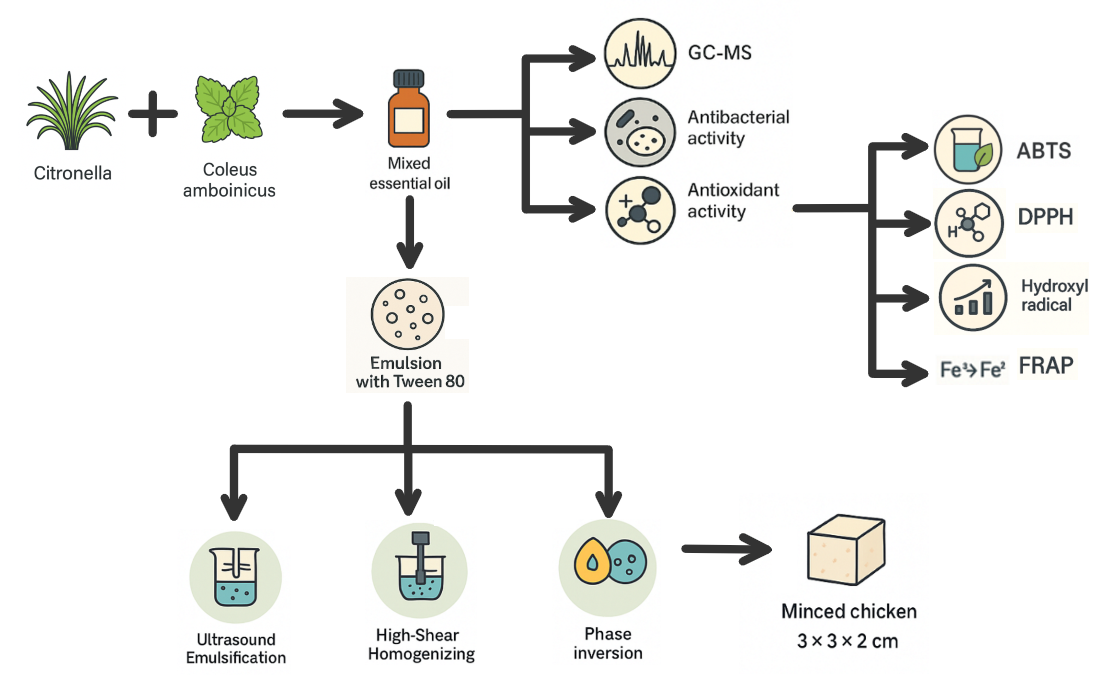

Supplement: S1 Fig — (TIFF) [file pone.0339984.s003.tiff]
